# Development of Novel Phase-Change Materials Derived from Methoxy Polyethylene Glycol and Aromatic Acyl Chlorides

**DOI:** 10.3390/polym15143069

**Published:** 2023-07-17

**Authors:** Alejandro Angel-López, Ángel Norambuena, C. Arriaza-Echanes, Claudio A. Terraza, Alain Tundidor-Camba, Deysma Coll, Pablo A. Ortiz

**Affiliations:** 1 Doctorado en Ciencias de Materiales Avanzados, Vicerrectoría de Investigación, Universidad Mayor, Santiago 8580745, Chile; alejandro.angel@mayor.cl (A.A.-L.); angel.norambuena@mayor.cl (Á.N.); c.arriazaechanes@gmail.com (C.A.-E.); 2 Instituto de Investigaciones y Control del Ejército de Chile (IDIC), Santiago 8370899, Chile; 3 Research Laboratory for Organic Polymers (RLOP), Department of Organic Chemistry, Pontificia Universidad Católica de Chile, Santiago 7820244, Chile; 4 Núcleo de Química y Bioquímica, Facultad de Ciencias, Ingeniería y Tecnología, Universidad Mayor, Santiago 8580745, Chile; 5 Centro de Nanotecnología Aplicada, Facultad de Ciencias, Ingeniería y Tecnología, Universidad Mayor, Santiago 8580745, Chile; 6 Escuela de Ingeniería en Medio Ambiente y Sustentabilidad, Facultad de Ciencias, Ingeniería y Tecnología, Universidad Mayor, Santiago 8580745, Chile

**Keywords:** phase-change material, thermal energy storage, methoxy polyethylene glycol, aromatic acyl chlorides

## Abstract

In this research, novel, organic, solid-liquid phase-change materials (PCMs) derived from methoxy polyethylene glycol (MPEG) and aromatic acyl chlorides (ACs) were prepared through a condensation reaction. The MPEGs were used as phase-change functional chains with different molecular weights (350, 550, 750, 2000, and 5000 g/mol). The aromatic ACs, terephthaloyl chloride (TPC) and isophthaloyl chloride (IPC), were employed as bulky linker cores. Solubility tests demonstrated that this family of PCMs is soluble in protic polar solvents such as H_2_O and MeOH, and insoluble in nonpolar solvents such as *n*-hexane. Fourier-ransform infrared spectroscopy (FT-IR UATR) and nuclear magnetic resonance (^1^H, ^13^C, DEPT 135°, COSY, HMQC, and HMBC NMR) were used to confirm the bonding of MPEG chains to ACs. The crystalline morphology of the synthesized materials was examined using polarized optical microscopy (POM), revealing the formation of spherulites with Maltese-cross-extinction patterns. Furthermore, it was confirmed that PCMs with higher molecular weights were crystalline at room temperature and exhibited an increased average spherulite size compared to their precursors. Thermal stability tests conducted through thermogravimetric analysis (TGA) indicated decomposition temperatures close to 400 °C for all PCMs. The phase-change properties were characterized by differential scanning calorimetry (DSC), revealing that the novel PCMs melted and crystallized between −23.7 and 60.2 °C and −39.9 and 45.9 °C, respectively. Moreover, the heat absorbed and released by the PCMs ranged from 57.9 to 198.8 J/g and 48.6 to 195.6 J/g, respectively. Additionally, the PCMs exhibited thermal stability after undergoing thermal cycles of melting-crystallization, indicating that energy absorption and release occurred at nearly constant temperatures. This study presents a new family of high-performance organic PCMs and demonstrates that the orientation of substituent groups in the phenylene ring influences supercooling, transition temperatures, and thermal energy storage capacity depending on the MPEG molecular weight.

## 1. Introduction

Currently, domestic hot water (DHW), and heating, ventilation, and air conditioning (HVAC) systems installed in commercial and residential buildings account for approximately 30% of the global energy consumption [1,2]. Moreover, the use of fossil fuels and electricity in HVAC systems, DHW systems, and the manufacturing of building materials contributes, directly and indirectly, to approximately one-third of the global greenhouse gas (GHG) emissions [3,4]. This, coupled with the depletion of energy resources and population growth [5], has led to an increase in the demand for energy sources and their costs, which have significantly risen in 2021 and 2022 [4,6]. On the other hand, this economic and energy expenditure is closely tied to the performance of the building’s envelope [7], which includes the walls, windows, roof, and floor. An inefficient envelope allows a continuous exchange of heat between the interior and exterior of the building, resulting in increased energy consumption [8]. Consequently, several research studies in the field of building energy efficiency emphasize the urgent need to enhance the energy performance of the envelope. This is to reduce energy consumption [9,10,11] and, incidentally, to reduce emissions of polluting gases using renewable energies such as solar thermal energy, characterized by its lack of GHG emissions [12].

Considering the abovementioned factors, thermal energy storage (TES) techniques have garnered significant interest due to their potential to integrate renewable energy sources into the global energy matrix. In addition, using TES systems optimizes solar thermal energy, enhances the energy efficiency of interconnected systems, and contributes to the reduction of environmental pollutants [13]. Among the materials commonly employed in TES systems, solid-liquid phase-change materials (PCMs) stand out [14]. These materials can store or release thermal energy during phase transitions within a limited temperature range [15].

Based on their chemical composition, PCMs can generally be classified into inorganic and organic categories. Compared to inorganic PCMs, such as salts and hydrated salts, organic PCMs exhibit more significant chemical and thermal stability and fewer issues related to supercooling and phase segregation [16]. Among organic PCMs, paraffin waxes (PWs) are noteworthy due to their high enthalpies of fusion and crystallization, which depend on the hydrocarbon chain length [17]. This means that increasing the number of carbon atoms in the PW chain leads to an increase in the melting temperature and enthalpy [18]. However, PWs do not exhibit congruent melting temperatures because they consist of a mixture of hydrocarbons with numerous isomers, which are difficult to purify and identify [19]. Additionally, PW are flammable materials with a pungent odor, which reduces human comfort [20]. Therefore, their encapsulation or support in other materials is essential for the development of TES applications [21,22,23].

PCMs are extensively used in various industries, including construction, telecommunications, electronics, food packaging, pharmaceuticals, and textiles, among others [24]. To effectively utilize a PCM, particularly in the building sector, it must exhibit a complete melting-crystallization cycle within the desired temperature range (working temperature), possess high enthalpies of fusion and crystallization, low supercooling, high chemical stability, and should not be corrosive or toxic. For efficient PCM application, three distinct temperature transition intervals have been recommended. These intervals include up to 21 °C in cooling applications, human thermal comfort between 22 and 28 °C, and water heating systems between 29 and 60 °C [25].

Among various organic PCMs, polyethylene glycol (PEG) and methoxy polyethylene glycol (MPEG) stand out due to their versatility, high enthalpy values of fusion and crystallization, a wide range of transition temperatures determined by their molecular weight [26], and their non-corrosive and non-toxic nature [27]. Additionally, the presence of hydroxyl end-groups in these polymers makes their structural modifications easy [28,29], developing new materials with diverse and improved properties. Several standard methods for obtaining PCMs based on PEG or MPEG involve physical mixing with high-porosity support materials [30] and chemical modification of the molecular structure to achieve solid-solid PCMs [31].

Among the various possibilities for linking two glycol chains, using ACs is considered one of the most effective strategies due to their high reactivity [32]. Ghaffari et al. [33] developed the synthesis and structural characterization of star-shaped macromolecules using trimesoyl chloride (TMC) as a trifunctional aromatic core and methoxy polyethylene glycol (MPEG) as polymeric chains with different molecular weights (350, 550, 750, and 2000 g/mol). Solubility tests on the star-shaped macromolecules revealed their insolubility in acetone and methanol, but solubility in water, chloroform, DMSO, and DMF. The authors concluded that TMC could be a suitable crosslinker for designing PEG-based crosslinked systems. In another study, Ö. Gök et al. [34] synthesized and structurally and thermally characterized new solid-liquid PCMs based on fatty alcohols, namely 1-dodecanol, 1-tetradecanol, and 1-octadecanol, as the phase-change chains, and terephthaloyl chloride (TPC) as the linking core to form fatty esters. Thermal testing showed that the PCMs melt between 66 and 81 °C, and the fusion enthalpies range from 179.2 to 191.2 J/g. This demonstrated that TPC fulfilled the role of a bulky bridging element between linear chains of fatty alcohols and increased the fusion temperature of these new materials. Moreover, the PCMs exhibited thermal stability during 1000 thermal cycles and displayed decomposition temperatures between 376 and 388 °C, allowing their application as organic PCMs at high temperatures.

As previously described, ACs enable the structural modification of glycols and alcohols, forming ester groups as linking agents characterized by low reactivity and corrosiveness [35]. Mainly, aromatic esters exhibit high stability due to their ability to conjugate with aromatic rings and stack into compact structures through π-π stacking interactions, resulting in structures with high fusion temperatures and great thermal stability when a large number of aromatic rings are clustered within the same macromolecule [36].

Considering these aspects, this study presents the synthesis of a group of materials that can act as organic PCMs, using MPEG as the phase-change functional chains with different molecular weights (350, 550, 750, 2000, and 5000 g/mol) and aromatic acyl dichlorides with different substituent group positions: terephthaloyl chloride (TPC) and isophthaloyl chloride (IPC). Ten novel phase-change materials (PCMs) were synthesized through condensation reactions, and their structures were characterized using spectroscopic techniques such as Fourier-transform infrared spectroscopy (FT-IR UATR) and nuclear magnetic resonance (^1^H, ^13^C, DEPT 135°, COSY, HMQC, and HMBC NMR). These techniques confirmed the formation of compounds derived from methoxy polyethylene glycol and aromatic acyl chlorides. The processability of the synthesized materials was evaluated in polar and nonpolar solvents through solubility tests. The thermal properties of the PCMs, including their phase-change temperatures and enthalpies, were analyzed using differential scanning calorimetry (DSC), while their thermal stability was assessed through thermogravimetric analysis (TGA). Additionally, DSC was used to perform ten thermal cycles on PCMs with transition temperatures at room temperature. Finally, the crystalline morphology of the PCMs was examined using polarized optical microscopy (POM). The novelty of this study is modifying the physicochemical properties of MPEG by including aromatic fragments in the main chain to modulate their properties based on the length of the phase-change functional chain and the orientation of substituent groups in the phenylene ring.

## 2. Materials and Experimental Methodology

### 2.1. Materials

Methoxy polyethylene glycol (MPEG, analytical grade, M_n_ = 350, 550, 750, 2000, and 5000 g/mol), triethylamine (TEA), methanol (MeOH), *n*-hexane, tetrahydrofuran (THF), chloroform (CHCl_3_), deuterated chloroform (CDCl_3_), terephthaloyl chloride (TPC; 203.02 g/mol), and isophthaloyl chloride (IPC, 203.02 g/mol) were obtained from Sigma-Aldrich (Milwaukee, WI, US). 

### 2.2. Characterization Techniques 

The synthesized PCMs’ molecular structures were confirmed by ^1^H, ^13^C, DEPT 135°, COSY, HMQC, and HMBC NMR spectroscopy using a Bruker AC-200 (200 and 400 MHz) spectrometer. Approximately 50 mg of the sample was dissolved in 0.5 mL of CDCl_3_. This procedure was repeated for the ten synthesized PCMs. FT-IR spectroscopy was performed on a Perkin Elmer Spectrum Two spectrophotometer with a UATR module (ZnSe), where approximately 10 mg of the sample was placed on the plate, completely covering the crystal. The measurement was performed in the range of 4000 to 400 cm^−1^ with a resolution of 0.5 cm^−1^.

For the solubility tests, 20 mg of the sample and 1.0 mL of the solvent (H_2_O, MeOH, *n*-hexane, CHCl_3_, and THF) were added to a glass test tube, shaken, and left to stand for ten minutes at room temperature. Samples were considered soluble in the tested solvent if they formed a homogeneous and transparent solution, partially soluble if they formed a turbid solution, and insoluble if they formed a heterogeneous solution with separate phases. 

The thermal stability of the PCMs was determined by thermogravimetric analysis (TGA) using a PerkinElmer TGA 4000 instrument. For this analysis, approximately 9.0 mg of PCMs were exposed from 20 °C to 600 °C at a heating rate of 10 °C/min and at a constant nitrogen flow rate of 20 mL/min.

Differential scanning calorimetry (DSC) determined the phase-change properties using a PerkinElmer DSC 4000 instrument. Approximately 7–13 mg of the sample was placed in aluminum sample pans and sealed. The measurements were performed in the temperature range from −70 °C to 100 °C at a heating rate of 1 °C/min, with a nitrogen flow rate of 20 mL/min. In the first scan, each sample was heated to 100 °C, held at this temperature for 1 min, and cooled to −70 °C to eliminate the thermal history of the PCM. In the second scan, which was recorded for analysis, the sample was heated again to 100 °C, held at this temperature for 1 min, and then cooled to −70 °C. For the analysis of thermal cycles by DSC, 7 mg of samples derived from MPEG750 g/mol were sealed in aluminum sample pans. In this case, the measurements were performed in the temperature range from −20 °C to 40 °C at the same heating rate and nitrogen flow rate described above (1 °C/min and 20 mL/min, respectively). In the first scan, each sample was heated to 40 °C, held at this temperature for 1 min, and cooled to −20 °C. In the next 10 scans, which were recorded for analysis, the sample was heated again to 40 °C, held at this temperature for 1 min, and then cooled to −20 °C. The melting and crystallization peaks were determined using Pyris^®^ thermal analysis software based on the tangent method, and the enthalpies were calculated by integrating the corresponding peaks.

The crystallinity of the PCMs was determined using polarized light optical microscopy (POM) on a Motic BA310Pol microscope equipped with a video camera and a 5× optical objective. For this purpose, approximately 25 mg of the sample was deposited on a glass slide, heated until complete fusion, and homogeneously dispersed on an approximately 900 mm^2^ surface. Immediately after, it was cooled at room temperature (20–25 °C) on an expanded polyethylene surface. Five photographs were taken per sample, four at the corners and one at the center. A count of well-defined crystalline domains was performed and averaged in each obtained image. Additionally, the distance between two opposite points located at the outermost part of each domain was measured, obtaining the average relative size.

### 2.3. PCMs’ Synthesis 

**Scheme 1:**
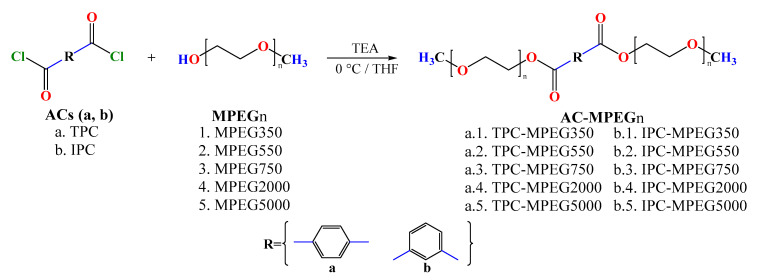
Synthesis route of phase-change materials derived from MPEG with molecular weights of (n) 350, 550, 750, 2000, or 5000 g/mol, and aromatics acyl chlorides (ACs) TPC and IPC.

The synthesis of the novel PCMs derived from MPEG and aromatic ACs was carried out by a previously reported technique, with slight modifications [33] (Scheme 1). The respective MPEG and TEA were dissolved in THF at room temperature in a three-necked flask. Subsequently, the system’s temperature was reduced to 0 °C in an ice-water bath, and the corresponding AC was added in an AC:MPEG ratio of 1:2. The amounts of materials used for the preparation of each PCM are shown in Table S1 in the Supplementary Material. The reaction was sustained with continuous stirring for 24 h under stationary nitrogen atmosphere (Figure S1 in Supplementary Material), because the constant flow of nitrogen extracts the solvent from the reaction medium. Then, the resulting mixture was filtered to remove the formed triethylammonium chloride, and the filtrate was concentrated to obtain the crude PCM. This PCM was dissolved in methanol (50 mL) and slowly poured onto *n*-hexane (100 mL), obtaining a white precipitate. The precipitate was isolated, dried, and then dissolved in THF to force the precipitation of any remaining triethylammonium chloride removed by filtration. The new filtrate was further concentrated to obtain a white solid, which was finally dried at 70 °C for 24 h.

For the PCMs using MPEG with molecular weights of 2000 and 5000 g/mol, the reaction was carried out in a THF:CHCl_3_ mixture (1:1 *v*/*v*) and heated to 60 °C to ensure complete dissolution of the precursors, before lowering the temperature to 0 °C.

**TPC-MPEG350**. Yield: 88%. FT-IR UATR (ZnSe, cm^−1^): 2940, 2870, 2818 (C-H, aliph); 1718 (C=O); 1271 (C-O, ester); 1096 (C-O, ether); 849 (*p*-subst). ^1^H-NMR (200 MHz, CDCl_3_, δ, ppm): 7.90 (s, 4H, **1**); 4.29 (m, 4H, **4**); 3.64 (m, 4H, **5**); 3.43 (m, 46H, **6**, **7**, **8**); 3.32 (m, 4H, **9**); 3.15 (s, 6H, **10**). ^13^C-NMR (200 MHz, CDCl_3_, δ, ppm): 165.1 (**3**); 133.5 (**2**); 129.1 (**1**); 71.4 (**9**); 70.1 (**6**, **7**, **8**); 68.6 (**5**); 64.1 (**4**); 58.5 (**10**). (Figure S2 in Supplementary Material).

**TPC-MPEG550**.Yield: 89%. FT-IR UATR (ZnSe, cm^−1^): 2938, 2868, 2821 (C-H, aliph); 1719 (C=O); 1272 (C-O, ester); 1096 (C-O, ether); 848 (*p*-subst). ^1^H-NMR (200 MHz, CDCl_3_, δ, ppm): 7.95 (s, 4H, **1**); 4.34 (m, 4H, **4**); 3.69 (m, 4H, **5**); 3.49 (m, 85H, **6**, **7**, **8**); 3.21 (s, 6H, **10**). ^13^C-NMR (200 MHz, CDCl_3_, δ, ppm): 165.3 (**3**); 133.6 (**2**); 129.3 (**1**); 71.6 (**9**); 70.2 (**6**, **7**, **8**,); 68.8 (**5**); 64.2 (**4**); 58.7 (**10**). (Figure S3 in Supplementary Material).

**TPC-MPEG750**. Yield: 92%. FT-IR UATR (ZnSe, cm^−1^): 2942, 2871, 2815 (C-H, aliph); 1716 (C=O); 1278 (C-O, ester); 1098 (C-O, ether); 841 (*p*-subst). ^1^H-NMR (200 MHz, CDCl_3_, δ, ppm): 8.04 (s, 4H, **1**); 4.43 (m, 4H, **4**); 3.78 (m, 4H, **5**); 3.57 (m, 124H, **6**, **7**, **8**); 3.47 (m, 4H, **9**); 3.30 (s, 6H, **10**). ^13^C-NMR (200 MHz, CDCl_3_, δ, ppm): 165.6 (**3**); 133.9 (**2**); 129.6 (**1**); 71.9 (**9**); 70.5 (**6**, **7**, **8**); 69.1 (**5**); 64.5 (**4**); 58.9 (**10**). (Figure S4 in Supplementary Material).

**TPC-MPEG2000**. Yield: 93%. FT-IR UATR (ZnSe, cm^−1^): 2948, 2882, 2810 (C-H, aliph); 1721 (C=O); 1278 (C-O, ester); 1099 (C-O, ether); 842 (*p*-subst). ^1^H-NMR (200 MHz, CDCl_3_, δ, ppm): 8.06 (s, 4H, **1**); 4.43 (m, 4H, **4**); 3.94 (m, 4H, **5**); 3.60 (m, 532H, **6**, **7**, **8**, **9**); 3.33 (s, 6H, **10**). ^13^C-NMR (200 MHz, CDCl_3_, δ, ppm): 165.8 (**3**); 133.9 (**2**); 129.6 (**1**); 72.0 (**9**); 70.6 (**6**, **7**, **8**); 69.1 (**5**); 64.5 (**4**); 59.0 (**10**). (Figure S5 in Supplementary Material).

**TPC-MPEG5000**. Yield: 92%. FT-IR UATR (ZnSe, cm^−1^): 2944, 2881, 2805 (C-H, aliph); 1721 (C=O); 1279 (C-O, ester); 1103 (C-O, ether); 841 (*p*-subst). ^1^H-NMR (200 MHz, CDCl_3_, δ, ppm): 8.04 (s, 4H, **1**); 4.44 (m, 4H, **4**); 3.93 (m, 4H, **5**); 3.58 (m, 1400H, **6**, **7**, **8**, **9**); 3.32 (s, 6H, **10**). ^13^C-NMR (200 MHz, CDCl_3_, δ, ppm): 162.2 (**3**); 133.2 (**2**); 129.5 (**1**); 71.9 (**9**); 70.5 (**6**, **7**, **8**); 69.1 (**5**); 64.5 (**4**); 59.0 (**10**). (Figure S6 in Supplementary Material).

**IPC-MPEG350**. Yield: 91%. FT-IR UATR (ZnSe, cm^−1^): 2949, 2869, 2813 (C-H, aliph); 1722 (C=O); 1238 (C-O, ester); 1095 (C-O, ether); 732 (*m*-subst). ^1^H-NMR (200 MHz, CDCl_3,_ δ, ppm): 8.61 (s, 1H, **1**); 8.15 (d, 2H, **2**); 7.44 (t, 1H, **3**); 4.41 (m, 4H, **6**); 3.76 (m, 4H, **7**); 3.55 (m, 50H, **8**, **9**, **10**); 3.45 (m, 4H, **11**); 3.28 (s, 6H, **12**). ^13^C-NMR (200 MHz, CDCl_3_, δ, ppm): 165.5 (**5**); 133.8 (**2**); 130.8 (**1**); 130.5 (**4**); 128.5 (**3**); 71.8 (**11**); 70.4 (**8**, **9**, **10**); 69.0 (**7**); 64.3 (**6**); 58.9 (**12**). (Figure S7 in Supplementary Material).

**IPC-MPEG550**.Yield: 93%. FT-IR UATR (ZnSe, cm^−1^): 2943, 2866, 2820 (C-H, aliph); 1723 (C=O); 1239 (C-O, ester); 1095 (C-O, ether); 733 (*m*-subst). ^1^H-NMR (200 MHz, CDCl_3,_ δ, ppm): 8.65 (s, 1H, **1**); 8.19 (d, 2H, **2**); 7.48 (t, 1H, **3**); 4.45 (m, 4H, **6**); 3.80 (m, 4H, **7**); 3.59 (m, 89H, **8**, **9**, **10**); 3.49 (m, 4H, **11**); 3.32 (s, 6H, **12**).^13^C-NMR (200 MHz, CDCl_3_, δ, ppm): 165.7 (**5**); 133.9 (**2**); 130.9 (**1**); 130.6 (**4**); 128.6 (**3**); 71.9 (**11**); 70.6 (**8**, **9**, **10**); 69.1 (**7**); 64.4 (**6**); 59.0 (**12**). (Figure S8 in Supplementary Material).

**IPC-MPEG750**. Yield: 91%. FT-IR UATR (ZnSe, cm^−1^): 2943, 2867, 2819 (C-H, aliph); 1723 (C=O); 1240 (C-O, ester); 1096 (C-O, ether); 734 (*m*-subst). ^1^H-NMR (200 MHz, CDCl_3_, δ, ppm): 8.64 (s, 1H, **1**); 8.18 (d, 2H, **2**); 7.48 (t, 1H, **3**); 4.45 (m, 4H, **6**); 3.79 (m, 4H, **7**); 3.58 (m, 125H, **8**, **9**, **10**); 3.48 (m, 4H, **11**); 3.32 (s, 6H, **12**). ^13^C-NMR (200 MHz, CDCl_3_, δ, ppm): 165.7 (**5**); 133.9 (**2**); 130.9 (**1**); 130.6 (**4**); 128.5 (**3**); 71.9 (**11**); 70.6 (**8**, **9**, **10**); 69.1 (**7**); 64.4 (**6**); 59.0 (**12**). (Figure S9 in Supplementary Material).

**IPC-MPEG2000**. Yield: 90%. FT-IR UATR (ZnSe, cm^−1^): 2944, 2882, 2803 (C-H, aliph); 1724 (C=O); 1238 (C-O, ester); 1101 (C-O, ether); 733 (*m*-subst). ^1^H-NMR (200 MHz, CDCl_3_, δ, ppm): 8.62 (s, 1H, **1**); 8.17 (d, 2H, **2**); 7.47 (t, 1H, **3**); 4.43 (m, 4H, **6**); 3.78 (m, 4H, **7**); 3.57 (m, 497H, **8**, **9**, **10**); 3.48 (m, 4H, **11**); 3.30 (s, 6H, **12**). ^13^C-NMR (200 MHz, CDCl_3_, δ, ppm): 165.6 (**5**); 133.7 (**2**); 130.9 (**1**); 130.7 (**4**); 128.5 (**3**); 71.9 (**11**); 70.5 (**8**, **9**, **10**); 69.1 (**7**); 64.4 (**6**); 59.0 (**12**). (Figure S10 in Supplementary Material).

**IPC-MPEG5000**. Yield: 90%. FT-IR UATR (ZnSe, cm^−1^): 2945, 2882, 2804 (C-H, aliph); 1723 (=O); 1238 (C-O, ester); 1103 (C-O, ether); 735 (*m*-subst). ^1^H-NMR (200 MHz, CDCl_3_, δ, ppm): 8.64 (s, 1H, **1**); 8.18 (d, 2H, **2**); 7.48 (t, 1H, **3**); 4.45 (m, 4H, **6**); 3.80 (m, 4H, **7**); 3.59 (m, 1773H, **8**, **9**, **10**); 3.41 (m, 4H, **11**); 3.32 (s, 6H, **12**). ^13^C-NMR (200 MHz, CDCl_3_, δ, ppm): 165.8 (**5**); 133.8 (**2**); 130.7 (**1**); 130.6 (**4**); 128.6 (**3**); 71.9 (**11**); 70.6 (**8**, **9**, **10**); 69.1 (**7**); 64.4 (**6**); 59.0 (**12**). (Figure S11 in Supplementary Material).

## 3. Results and Discussion

### 3.1. PCMs’ Synthesis and Spectroscopy Characterization

**Figure 1:**
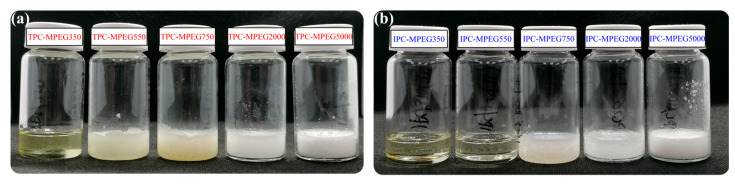
Images of TPC-MPEG (**a**) and IPC-MPEG (**b**) series at room temperature (20–25 °C).

To finally purify the novel PCMs derived from MPEG and ACs, they were dissolved in THF and filtered through a 3.1 μm glass fiber filter to remove traces of residual triethylammonium chloride. Subsequently, they were concentrated and dried at 70 °C to obtain the TPC-MPEG and IPC-MPEG series shown in Figure 1, which were further characterized both structurally and thermally. 

**Figure 2:**
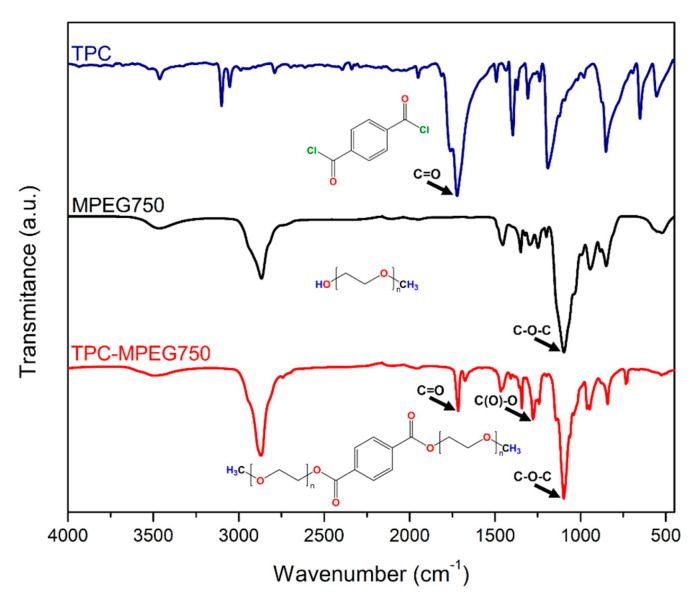
FT-IR spectra of terephthaloyl chloride (TPC, blue line), methoxy polyethylene glycol 750 g/mol (MPEG750, black line), and TPC-MPEG750 (red line).

Figure 2 presents the FT-IR spectra of TPC, MPEG750, and TPC-MPEG750 as an example of the characterization of the synthesized PCMs. A comparison of the three spectra allowed us to confirm the presence of the novel PCMs. In the first spectrum, TPC (blue line) bands corresponding to the C-H stretching of the phenyl ring were observed at 3101 and 3053 cm^−1^ [37], along with carbonyl (C=O) bands at 1742 and 1722 cm^−1^ [38]. Additionally, a band at 850 cm^−1^ indicated the presence of the *p*-substituted ring [39]. The spectrum of the MPEG750 precursor (black line) exhibited an O-H stretching band at 3470 cm^−1^ and C-H stretching bands at 2942, 2866, and 2816 cm^−1^ coming from the rest of the chain [40]. The vibration band of the C-O-C linkage corresponding to the ether groups in the repeating unit of the polymer was observed at 1098 cm^−1^. Comparing these results with those obtained from the TPC-MPEG750 reaction product (red line), only one band corresponding to the carbonyl (C=O) stretching was observed at 1716 cm^−1^ [41]. This indicated the functional group change resulting from the substitution of chlorine by the MPEG used. This result, supported by the presence of the C(O)-O stretching at 1278 cm^−1^, indicated the formation of ester groups [42]. Additionally, the band at 841 cm^−1^ confirmed the presence of the *p*-substituted phenyl ring derived from the TPC precursor. Furthermore, bands at 1098 cm^−1^ and 2870 cm^−1^ corresponded to the C-O-C linkage and C-H stretching [43], respectively, both from MPEG750. This procedure was analogously performed for the IPC-derived series, as shown in Figure S12 of the Supplementary Material.

**Figure 3:**
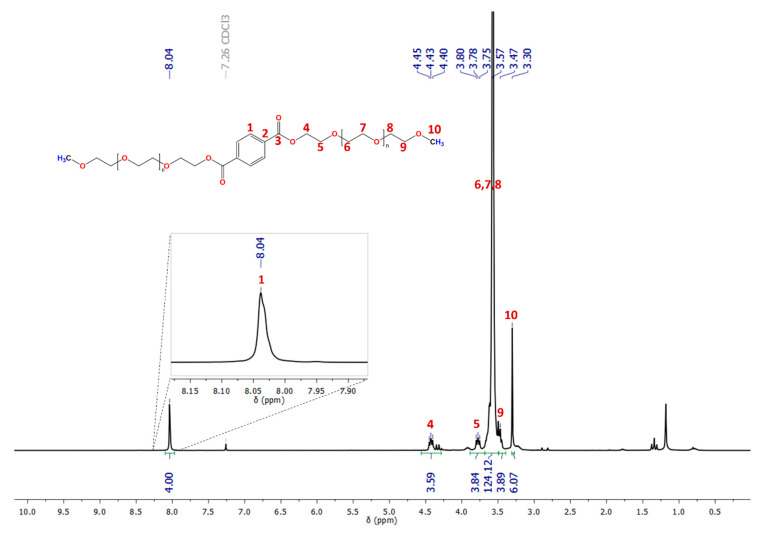
^1^H NMR spectra of TPC-MPEG750.

Figure 3 represents an example of the ^1^H NMR spectroscopic analysis used to confirm the synthesis of the designed PCMs. The ^1^H NMR spectrum of purified TPC-MPEG750 dissolved in CDCl_3_ is shown in this case. The first significant signal observed was a singlet at 8.04 ppm, integrating for 4, which was attributed to the four protons of the phenyl ring (H-1). In the region where aliphatic hydrogens are typically observed, two multiplets at 4.43 ppm and 3.78 ppm, both integrating for 4 (H-4 and H-5), were observed. These signals were assigned to the methylene hydrogens closest to the ester group, which experienced greater de-shielding than the other methylenes due to their proximity to the ester group. The intense multiplet at 3.57 ppm, integrating for 124 (H-6, H-7, and H-8), was attributed to the methylene hydrogens from the repeating unit of MPEG750, which are not near the ester group. Furthermore, the multiplet at 3.47 ppm, integrating for 4 (H-9), was assigned to the methylene hydrogens adjacent to the methoxy group. Finally, a singlet at 3.30 ppm, integrating for 6, was identified and assigned to the six methyl hydrogens from MPEG750 (H-10). These signals and their integrals are consistent with similar structures reported by other research studies [33,34].

Due to the number of methylene hydrogens present and the polydispersity of each MPEG, a comparison was performed between the hydrogens contributed by each MPEG and the resulting integrals. For this purpose, the expected integral was calculated using Equation (1), where *M_n_* means the average molecular weight of the used MPEG, *m_OH_* means the present hydroxyl, *m_CH_*_3_ represents the terminal methyl, and *M_u_* represents the molecular weight of the respective repeating unit (-CH_2_CH_2_O-). This calculation resulted in the methylenes’ theoretical integral (*I*_t_) in each synthesized PCM.

(1) $$I_{t}=4\left[\frac{\;M_{n}-m_{OH}-m_{CH3}}{M_{u}}\right]$$

For TPC-MPEG750, the approximate *I_t_* was 130. This value closely matched the 136 hydrogens obtained by integrating the signals at 4.43, 3.78, 3.57, and 3.47 ppm (Figure 3), which represent all the methylene hydrogens present in each PCM (Table 1). This confirms that the MPEG was connected on both sides of the aromatic ring through the ester group. The same applied to the IPC-MPEG series (see Supplementary Material Table S2).

**Figure 4:**
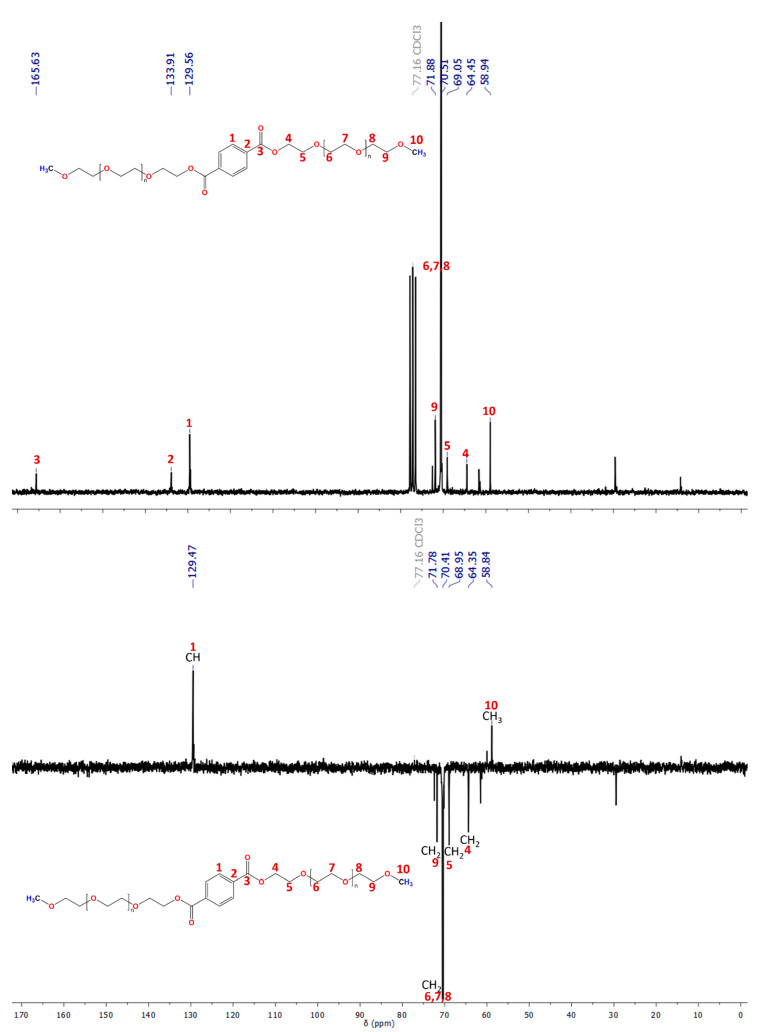
^13^C NMR and DEPT-135° spectra of TPC-MPEG750.

Figure 4 shows an example of the ^13^C NMR and DEPT-135° spectra of TPC-MPEG750, performed in CDCl_3_ as the solvent. In the ^13^C spectrum, the most de-shielded carbon from the ester group formed (C-3) was observed at 165.6 ppm. In the region where aromatic carbons are typically observed, two signals were observed at 133.9 ppm and 129.6 ppm. The first signal corresponded to the two quaternary carbons of the aromatic ring adjacent to the ester group (C-1), and the second signal corresponded to the remaining four equivalent carbons of the phenyl ring (C-2). The methylene carbons adjacent to the methoxy group (C-9) were observed at 71.9 ppm. Meanwhile, the highest-intensity signal at 70.5 ppm was assigned to the methylene carbons in the repeating unit (C-6, C-7, and C-8) originating from MPEG. Furthermore, at higher fields, the methylene carbons adjacent to the ester group were observed at 69.1 ppm and 64.5 ppm (C-5 and C-4), and the carbon of the terminal methoxy group was observed at 58.9 ppm (C-10). The assignment of C-1 as the carbon of the aromatic ring and C-10 as the terminal methyl carbon was confirmed by comparing the ^13^C spectrum with the DEPT-135° spectrum. Likewise, the assignment of carbons C-4 to C-9 as methylene carbons was confirmed by NMR spectra such as COSY, HMQC, and HMBC (see Supplementary Material Figure S7).

### 3.2. Solubility Tests

To determine the most suitable solvent for processing and/or application purposes, solubility assays were assessed with the previously purified and spectroscopically characterized PCMs. Table 2 shows the solubility of PCMs (IPCMPEG and TPC-MPEG series) at room temperature in different solvents. It was observed that both series exhibited the same behavior towards the assessed solvents. These results revealed that synthesized PCMs are soluble in polar protic solvents such as H_2_O and MeOH due to their feasibility of developing hydrogen bonds with the PCMs’ ether groups. It was also observed that PCMs with shorter chains (MPEG350-750) were soluble in THF, while those PCMs with larger chains (MPEG2000-5000) were insoluble at room temperature and required heat for complete dissolution. Moreover, both series were soluble in CHCl_3_ and in a THF: CHCl_3_ mixture (1:1 *v*/*v*). Lastly, it was determined that PCMs were insoluble in nonpolar solvents such as *n*-hexane.

### 3.3. PCMs’ Thermal Properties

**Figure 5:**
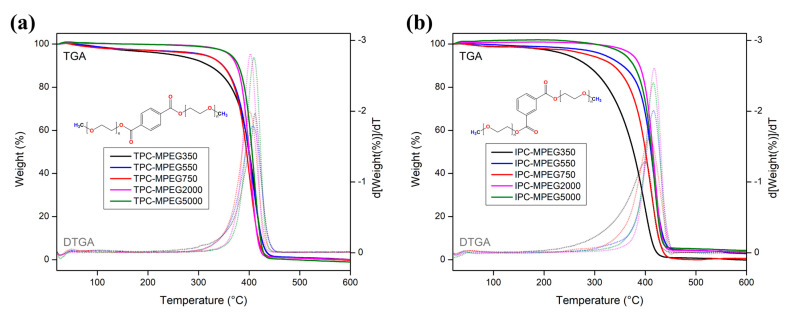
TGA thermograms obtained of the TPC-MPEG (**a**) and IPC-MPEG (**b**) series.

Thermogravimetric analyses were conducted on the synthesized PMCs to assess the materials’ thermal stability in function of temperature. The results extracted from the thermograms obtained for each PCM (Figure 5) are summarized in Table 3. It was observed that all PCMs initiated degradation (T_i_) over 180 °C, which is above the application temperature of these PCMs. The values of 5% and 10% weight loss degradation temperature (T_d5%_ and T_d10%_) showed a progressive increase as the size of the MPEG segment used increased. This was due to the larger size of the MPEG-derived segment where degradation was expected to start. However, all the synthesized PCMs were rapidly degraded at temperatures of around 400 °C (T_d_), the same applied with similar chemical structures of PCMs [34] and different PCMs that used polyoxyethylenes as phase-change chains [44,45]. This demonstrates that at this temperature, sufficient energy was provided to initiate the total degradation of each PCM. Contrary to expectations, theoretically, PCMs with a smaller flexible segment would exhibit a more outstanding contribution of aromatic content to thermal stability; however, the aromatic content of PCMs did not enhance the thermal stability at degradation temperatures around 400 °C. This suggests that degradation started in the region derived from MPEG, and once it reached the aromatic region (central ring), it continued until complete degradation of the material was achieved. This phenomenon was observed in the residues obtained after each analysis. PCM samples with larger lengths left less than 4.3% of residue, which could be reduced to 0% if the analysis was conducted until 900 °C. 

**Figure 6:**
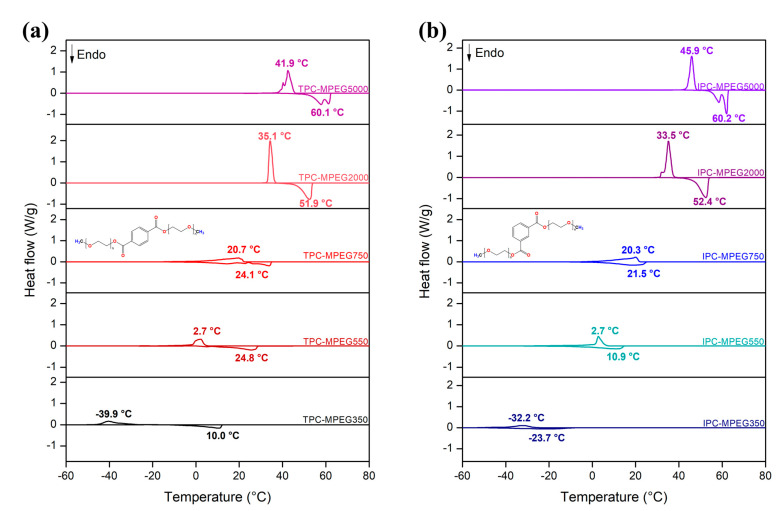
Thermograms obtained from DSC analyses of the TPC-MPEG (**a**) and IPC-MPEG (**b**) series.

Once the thermogravimetric analyses were completed and the decomposition temperatures of the PCMs were determined, calorimetric analysis was performed to determine the temperatures and energies associated with the melting and crystallization process of the PCM (Figure 6). Table 4 summarizes the results of the calorimetric assays, the estimated average molecular weight of the PCMs (M_n_), and the percentage of aromatic content (A_r_) with respect to M_n_; where, M_n_ is calculated as the sum of the aromatic AC’s molecular weight (without considering the chlorine atoms) and the molecular weight of the two MPEG chains reported by the supplier (without considering the hydrogen atoms of the terminal hydroxyls) [46,47,48,49,50].

It can be observed that both the IPC-derived series and the TPC series showed a progressive increase in the values of melting (T_m_) and crystallization (T_c_) temperatures. In both cases, this increase was proportional to the length of the linear chain segment derived from the different MPEGs used, similar to what occurs with paraffinic-type PCMs [51]. As the chain length increased, the contact surface area between the chains also increased, enhancing the magnitude of the intermolecular forces. Consequently, a greater amount of energy was required to transition from one phase to another.

When comparing the respective values of T_m_ and T_c_ for the IPC series with their counterparts in the TPC series, it was observed that the PCMs derived from MPEG350, 550, and 750 in the TPC series exhibited higher values for these temperatures. Conversely, these values were similar for PCMs in both series synthesized from MPEG2000 and 5000. Two important conclusions can be drawn from this. First, when the aromatic fraction was below 4% by mass, its influence on the thermal properties became negligible for these types of PCMs. Second, the orientation of the substituent groups on the aromatic ring generated a difference in the molecular ordering of PCMs. This phenomenon has been widely reported in analyzing the thermal properties of polymers that use these kinds of aromatic groups. Those with *p*-substitution of the aromatic rings exhibited higher ordering and greater packing than those with *m*-substitution [52]. This caused higher transition temperatures due to the increased magnitude of intermolecular forces.

Another critical parameter to analyze is the difference between the T_m_ and T_c_ temperatures of the PCMs, which is defined as one of the recurring challenges for a PCM, known as supercooling [53]. By examining the difference between these temperatures in both series, it was observed that the PCMs derived from MPEG750 in both series showed lower supercooling values compared to the other PCMs. For a substance to crystallize, it must be arranged and oriented to form the respective unit cells that give rise to the solid states. In the case of large molecules such as polymers, due to their length, a significant amount of time was required for this process to occur, often making it extremely slow. This explains why PCMs derived from MPEG2000 and 5000 exhibited differences of around 14 °C. On the other hand, the PCMs derived from MPEG350 and 550 showed values ranging from 8 to 50 °C. This difference was attributed to the aromatic ring located in the central part of the PCM’s molecular structure, which, due to its extensive length, broke the symmetry of the chain, requiring lower temperatures as the length of the linear region decreased.

Similarly to T_m_ and T_c_, the enthalpies of their respective transitions showed a proportional increase with the length of the linear segment [54], complementing the idea that an increase in the contact surface enhances the magnitude of intermolecular forces and, consequently, the energy required to transition between phases. Likewise, it was observed that the TPC series needed more energy for the transitions due to the enhanced packing provided by the *p*-substituted ring. However, unlike T_m_ and T_c_, the enthalpies did not show a noticeable dependence on the percentage of aromatic content. This could be attributed to the molecular ordering of the materials and requires further investigation. 

**Figure 7:**
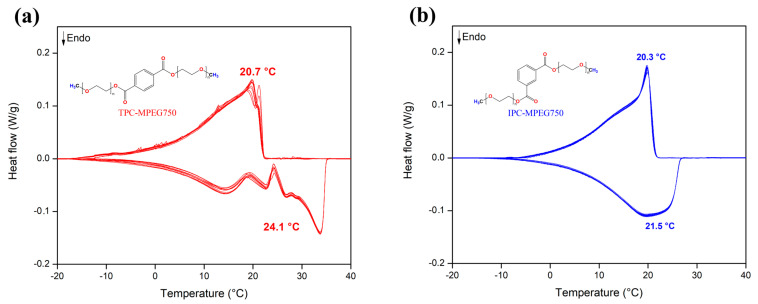
Ten thermal cycles in the DSC of TPC-MPEG750 (**a**) and IPC-MPEG750 (**b**).

Once the melting and crystallization temperatures and their respective enthalpies were determined, the influence of prolonged heat cycling on these properties was investigated. This was accomplished by repetitively subjecting two PCMs (TPC-MPEG750 and IPC-MPEG750) to a confined temperature range. These PCM selections were based on their T_m_ and T_c_ values closest to the operating temperature. Both PCMs underwent ten thermal cycles, including the melting and crystallization processes, as shown in Figure 7. The results of all the analyses conducted on the two PCMs are summarized in Table 5.

For the IPC-MPEG750 sample, slight variations in the melting and crystallization temperatures were observed. This indicates that energy absorption and release occurred at nearly constant temperatures. The same is true for the melting and crystallization enthalpies. This demonstrated no significant variations and indicated thermal stability during the thermal cycling process. However, it is necessary to increase the number of thermal cycles to 100 cycles to confirm this behavior and ensure reliable performance during extended operational periods [45,55].

### 3.4. PCMs’ Crystal Morphology

**Figure 8:**
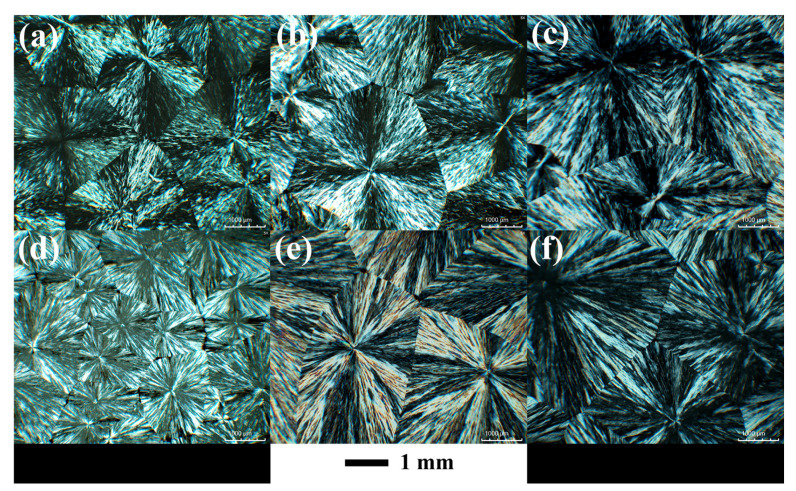
POM images (5×) of MPEG2000 (**a**), TPC-MPEG2000 (**b**), IPC-MPEG2000 (**c**), MPEG5000 (**d**), TPC-MPEG5000 (**e**), and IPC-MPEG5000 (**f**).

The crystalline morphology of the novel synthesized PCMs was observed using polarized optical microscopy (POM). This analysis could only be conducted for PCMs in a solid state at room temperature (derivatives of MPEG2000 and 5000 g/mol series). Although the PCMs derived from MPEG750 had crystallization temperatures close to room temperature, when they were placed under the microscope’s light, the heat emitted by it was sufficient to cause PCM melting, making observation of its crystal structure impossible. Figure 8 shows the crystal structure of the MPEG precursor and its respective derivatives, where typical Maltese-cross-extinction patterns of spherulites under POM were clearly exhibited in all PCMs. Additionally, it was possible to observe that the MPEG5000 derivatives showed an increase in the average size of spherulites, with dimensions of 2.0 mm for MPEG5000 and 3.2 mm and 3.3 mm for its derivatives (see Table 6). This means that fewer spherulites were observed in an equivalent surface area. This result was also observed for the derivatives of MPEG2000, indicating that an increase in molecular size leads to the formation of larger agglomerates and, consequently, larger spherulites.

## 4. Conclusions

Successfully synthesized novel phase-change materials were obtained through a condensation reaction between MPEGs and acyl chlorides (TPC and IPC). Characterization techniques, including FT-IR and ^1^H, ^13^C, dept 135°, COSY, HMQC, and HMBC NMR analyses, confirmed the molecular structure of the proposed PCMs. Solubility tests demonstrated that all PCMs were soluble in polar protic solvents such as H_2_O, MeOH, and CHCl_3_, while they were insoluble in nonpolar solvents such as *n*-hexane.

Thermal stability analysis revealed that all PCMs initiated degradation (T_i_) at around 180 °C, and complete degradation (T_d_) occurred at approximately 400 °C. However, the aromatic content did not significantly affect the thermal stability of the PCMs, exhibiting similar values to pure MPEGs.

Regarding the phase-change properties, the melting and crystallization temperatures ranged from −23.7 to 60.2 °C and −39.9 to 45.9 °C, respectively. These results suggest potential applications of PCMs in the residential and industrial building sector, including refrigeration (TPC-MPEG350, IPC-MPEG350, and IPC-MPEG550), human thermal comfort (TPC-MPEG550, TPC-MPEG750, and IPC-MPEG750), and hot water systems (TPC-MPEG2000, TPC-MPEG5000, IPC-MPEG2000, and IPC-MPEG5000). 

The absorbed and released heat by the PCMs ranged from 57.9 to 198.8 J/g and 48.6 to 195.6 J/g, respectively, showing that it is possible to increase its value with the molecular weight of MPEG. 

Furthermore, the results indicated that when the aromatic fraction was below 4% by mass, it had a negligible influence on the thermal properties of the synthesized PCMs. The orientation of the substituent groups on the aromatic ring affected molecular ordering and, therefore, the transition temperatures. Finally, the observed supercooling values between 1.2 °C and 49.9 °C demonstrated the possibility of significantly reducing supercooling by modifying the chain length of these PCMs and their orientation relative to the central aromatic group.

These findings offer new perspectives for designing and developing phase-change materials with adjustable properties, contributing to a better understanding of how the molecular structure affects the thermal properties of PCMs and enabling optimization for applications in the construction industry. To sum up, this study provided a solid foundation of organic PCMs’ development and application based on aromatic acyl chlorides and methoxy polyethylene glycol, opening new opportunities in the field of phase-change materials for various applications in DHW and HVAC building systems. 

## Figures and Tables

**Table 1:** Theoretical hydrogen counts (*I_t_*) calculated in the repeating unit, and hydrogen counts obtained by ^1^H NMR in the TPC-MPEG series.

Signal Number	δ (ppm) and Integral (*I*)	PCMs				
		TPC- MPEG350	TPC- MPEG550	TPC- MPEG750	TPC- MPEG2000	TPC- MPEG5000
1	δ	7.90	7.95	8.04	8.10	8.04
	*I*	4	4	4	4	4
4	δ	4.29	4.34	4.43	4.49	4.44
	*I*	4	4	4	4	4
5	δ	3.64	3.69	3.78	3.99	4.12
	*I*	4	4	4	4	4
6,7,8	δ	3.43	3.49	3.57	3.64	3.58
	*I*	46	85	124	532	1400
9	δ	3.32	3.38	3.47	- ^a^	- ^a^
	*I*	4	4	4	- ^a^	- ^a^
10	δ	3.15	3.21	3.30	3.37	3.32
	*I*	6	6	6	6	6
Integral	$\sum_{4}^{9}I_{n}$	58	97	136	540	1408
	*I_t_*	58	94	130	357	902

δ: Chemical shift in ^1^H NMR spectrum, *I*: integral obtained by ^1^H NMR, *I_t_*: integral obtained by Equation (1), $\overset{9}{\underset{4}{\sum }}I_{n}$: summation of the integrals of signals 4 to 9 obtained by ^1^H NMR, and ^a^: signal 9 is overlapped with signals 6, 7, and 8 (see Supplementary Material, Figures S5 and S6).

**Table 2:** Solubility values of the obtained PCMs.

Solvents	^a^ IPC-				
	MPEG350	MPEG550	MPEG750	MPEG2000	MPEG5000
H_2_O	+/−	+/−	+/−	+/−	+/−
MeOH	+	+	+	+	+
*n*-hexane	−	−	−	−	−
CHCl_3_	+	+	+	+	+
THF	+	+	+	−	−
THF/CHCl_3_	+	+	+	+	+
**Solvents**	^**a**^ **TPC-**				
	**MPEG350**	**MPEG550**	**MPEG750**	**MPEG2000**	**MPEG5000**
H_2_O	+/−	+/−	+/−	+/−	+/−
MeOH	+	+	+	+	+
*n*-hexane	−	−	−	−	−
CHCl_3_	+	+	+	+	+
THF	+	+	+	−	−
THF/CHCl_3_	+	+	+	+	+

^a^ [C] = 20 mg in 1.0 mL of solvent, + soluble at room temperature (RT), +/− partially soluble at RT, and − insoluble at RT.

**Table 3:** Thermal parameters of PCMs obtained by TGA: MPEG, TPC-MPEG, and IPC-MPEG series.

PCMs	T_i_	T_onset_	T_d_	T_d5%_	T_d10%_	R
	(°C)	(°C)	(°C)	(°C)	(°C)	(%)
MPEG350	179.0	258.6	337.9	204.5	236.2	0.0
MPEG550	231.5	339.4	385.7	243.2	297.5	0.0
MPEG750	267.3	370.7	401.4	171.8	269.4	0.0
MPEG2000	329.0	389.1	405.1	380.9	394.4	0.9
MPEG5000	334.7	392.6	406.3	392.5	400.4	1.2
TPC-MPEG350	238.0	365.3	399.2	250.8	321.4	0.1
TPC-MPEG550	285.0	363.3	398.1	311.6	348.4	0.0
TPC-MPEG750	270.0	361.1	392.4	309.2	347.9	0.0
TPC-MPEG2000	292.0	376.5	398.1	361.6	375.1	0.1
TPC-MPEG5000	294.0	376.7	398.7	363.5	378.8	0.0
IPC-MPEG350	183.0	328.7	387.0	245.5	286.5	0.0
IPC-MPEG550	252.0	373.2	403.9	318.0	355.3	2.9
IPC-MPEG750	248.0	359.5	396.1	287.8	337.1	0.6
IPC-MPEG2000	2630	382.0	406.2	374.3	388.8	3.7
IPC-MPEG5000	250.0	377.5	404.1	359.8	377.4	4.3

T_i_: Degradation initial temperature, T_onset_: thermal effect onset temperature, T_d_: maximum decomposition rate temperature, T_d5%_: 5% weight loss degradation temperature, T_d10%_: 10% weight loss degradation temperature, and R: residue percentage.

**Table 4:** Summary of the thermal properties of the PCMs determined by DSC.

PCM	M_n_ (g/mol)	A_r_ (%)	Melting Process		Crystallization Process		Supercooling
			T_m_ (°C)	ΔH_m_ (J/g)	T_c_ (°C)	ΔH_c_ (J/g)	ΔT (°C)
TPC-MPEG350	830	9.2	10.0	79.8	−39.9	64.6	49.9
TPC-MPEG550	1230	6.2	24.8	100.6	2.7	75.8	22.1
TPC-MPEG750	1630	4.7	24.1	111.2	20.7	109.6	3.4
TPC-MPEG2000	4130	1.8	51.9	185.8	35.1	183.7	16.8
TPC-MPEG5000	10,130	0.8	60.1	164.0	41.9	157.6	18.2
IPC-MPEG350	830	9.2	−23.7	57.9	−32.2	48.6	8.5
IPC-MPEG550	1230	6.2	10.9	98.4	2.7	81.0	8.2
IPC-MPEG750	1630	4.7	21.5	99.1	20.3	87.0	1.2
IPC-MPEG2000	4130	1.8	52.4	198.8	33.5	195.6	18.9
IPC-MPEG5000	10,130	0.8	60.2	169.8	45.9	171.5	14.3

M_n_: Estimated average molecular weight, A_r_: aromatic content, T_m_: melting temperature, ΔH_m_: melting enthalpy, T_c_: crystallization temperature, ΔH_c_: crystallization enthalpy, and ΔT: supercooling.

**Table 5:** TPC-MPEG750 and IPC-MPEG750 thermal properties.

Cycle	TPC-MPEG750 Thermal Properties				
	T_m_ (°C)	ΔH_m_ (J/g)	T_c_ (°C)	ΔH_c_ (J/g)	ΔT (°C)
1	23.8	113.3	20.2	108.9	3.5
2	23.9	101.6	22.5	119.3	1.4
3	24.0	107.0	22.5	104.6	1.5
4	24.1	119.0	20.1	103.9	4.0
5	24.2	120.5	20.1	113.2	4.0
6	24.2	117.2	20.2	110.4	4.0
7	24.2	119.0	20.2	101.1	3.9
8	24.2	111.4	20.2	105.3	4.0
9	24.3	102.0	20.3	115.8	4.0
10	24.3	101.4	20.3	113.1	4.1
**Cycle**	**IPC-MPEG750 Thermal Properties**				
	**T_m_ (°C)**	**ΔH_m_ (J/g)**	**T_c_ (°C)**	**ΔH_c_ (J/g)**	**ΔT (°C)**
1	21.4	100.6	20.3	88.3	1.1
2	21.5	99.4	20.3	86.6	1.1
3	21.5	100.9	20.3	89.0	1.2
4	21.5	100.8	20.3	89.3	1.2
5	21.5	99.7	20.3	88.4	1.2
6	21.5	99.0	20.3	87.0	1.2
7	21.4	98.1	20.3	85.1	1.2
8	21.4	98.0	20.3	84.6	1.2
9	21.4	96.5	20.3	84.6	1.1
10	21.4	98.4	20.3	87.4	1.2

T_m_: Melting temperature, ΔH_m_: melting enthalpy, T_c_: crystallization temperature, ΔH_c_: crystallization enthalpy, and ΔT: supercooling.

**Table 6:** Quantity and mean size of spherulites.

PCM	Quantity	Mean Size (mm)	σ (mm)
MPEG2000	8	2.5	0.4
TPC-MPEG2000	7	2.6	0.6
IPC-MPEG2000	4	3.8	0.7
MPEG5000	14	2.0	0.3
TPC-MPEG5000	5	3.2	0.6
IPC-MPEG5000	4	3.3	0.6

σ: Standard deviation.

## Data Availability

Data is contained within the article or Supplementary Material.

## Supplementary Materials

The following supporting information can be downloaded at: https://www.mdpi.com/article/10.3390/polym15143069/s1, Figure S1: Experimental setup used for the synthesis of PCMs. Table S1: Amount of the used materials for the preparation of each PCM. Figure S2: Infrared and 1H, 13C, and DEPT 135° NMR spectra of TPC-MPEG350. Figure S3: Infrared and 1H, 13C, and DEPT 135° NMR spectra of TPC-MPEG550. Figure S4: Infrared and 1H, 13C, and DEPT 135° NMR spectra of TPC-MPEG750. Figure S5: Infrared and 1H, 13C, and DEPT 135° NMR spectra of TPC-MPEG2000. Figure S6: Infrared and 1H, 13C, and DEPT 135° NMR spectra of TPC-MPEG5000. Figure S7: Infrared and 1H, 13C, DEPT 135°, COSY, HMQC, and HMBC NMR spectra of IPC-MPEG350. Figure S8: Infrared and 1H, 13C, and DEPT 135° NMR spectra of IPC-MPEG550. Figure S9: Infrared and 1H, 13C, and DEPT 135° NMR spectra of IPC-MPEG750. Figure S10: Infrared and 1H, 13C, and DEPT 135° NMR spectra of IPC-MPEG2000. Figure S11: Infrared and 1H, 13C, and DEPT 135° NMR spectra of IPC-MPEG5000. Figure S12: Comparison of IPC, MPEG750, and IPC-MPEG750 infrared spectra. Table S2: Theoretical hydrogen counts (It) calculated in the repeating unit, and hydrogen counts obtained by 1H NMR in the IPC-MPEG series.
