# Supplementary material for: Development of Novel Phase-Change Materials Derived from Methoxy Polyethylene Glycol and Aromatic Acyl Chlorides
Source: Polymers (Basel). 2023 Jul 17;15(14):3069. doi: 10.3390/polym15143069 (PMC10384409; doi:10.3390/polym15143069)
Supplement: Supplementary file 1 [file polymers-15-03069-s001.zip › polymers-2467835-supplementary.pdf]

Development of Novel Phase-Change Materials Derived from Methoxy Polyethylene Glycol and Aromatic Acyl Chlorides

Alejandro Angel-López [1], Ángel Norambuena [1,2], C. Arriaza-Echanes [1], Claudio A. Terraza [3], Alain Tundidor-Camba [3], Deysma Coll [4,5] and Pablo A. Ortiz [5,6,*]

1 Doctorado en Ciencias de Materiales Avanzados, Vicerrectoría de Investigación, Universidad Mayor, Santiago 8580745, Chile; alejandro.angel@mayor.cl (A.A.-L.); angel.norambuena@mayor.cl (Á.N.); c.arriazaechanes@gmail.com (C.A.-E.)
2 Instituto de Investigaciones y Control del Ejército de Chile (IDIC), Santiago 8370899, Chile
3 Research Laboratory for Organic Polymers (RLOP), Department of Organic Chemistry, Pontificia Universidad Católica de Chile, Santiago 7820244, Chile; cterraza@uc.cl (C.A.T.); atundido@uc.cl (A.T.-C.)
4 Núcleo de Química y Bioquímica, Facultad de Ciencias, Ingeniería y Tecnología, Universidad Mayor, Santiago 8580745, Chile; deysma.coll@umayor.cl
5 Centro de Nanotecnología Aplicada, Facultad de Ciencias, Ingeniería y Tecnología, Universidad Mayor, Santiago 8580745, Chile
6 Escuela de Ingeniería en Medio Ambiente y Sustentabilidad, Facultad de Ciencias, Ingeniería y Tecnología, Universidad Mayor, Santiago 8580745, Chile
* Correspondence: pablo.ortiz@umayor.cl

SUPPLEMENTARY MATERIAL

1. Experimental methodology

Figure S1 shows the experimental setup used during the synthesis of the PCMs derived from the methoxy polyethylene glycol and aromatic acyl chlorides.

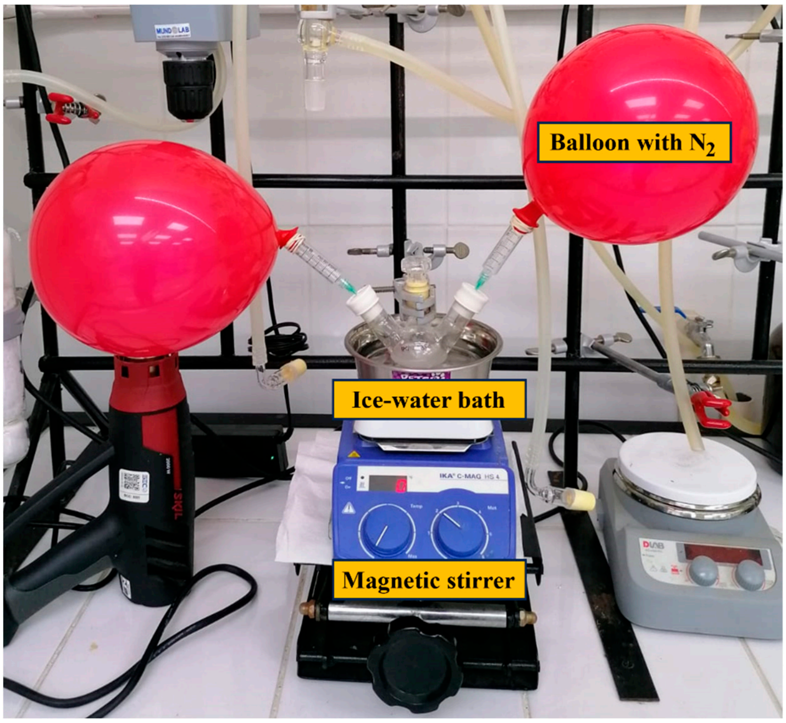

Figure S1. Experimental setup used for the synthesis of PCMs.

2. PCMs synthesis

Table S1 shows the amounts of the used materials for the preparation of each sample.

Table S1. Amount of the used materials for the preparation of each PCM.

**PCMs**	**MPEG (mmol)**	**TEA (mmol)**	**THF (mL)**	**$CHCl_3$ (mL)**	**AC (mmol)**	**Yield (%)**
TPC-MPEG350	12.2	12.2	10.0	0.0	6.0	88.1
TPC-MPEG550	8.2	8.2	10.0	0.0	4.1	89.0
TPC-MPEG750	6.2	6.2	10.0	0.0	3.1	91.7
TPC-MPEG2000	2.5	2.5	5.0	5.0	1.2	92.8
TPC-MPEG5000	1.0	1.0	5.0	5.0	0.5	92.3
IPC-MPEG350	12.2	12.2	10.0	0.0	6.0	90.5
IPC-MPEG550	8.2	8.2	10.0	0.0	4.1	93.4
IPC-MPEG750	6.2	6.2	10.0	0.0	3.1	91.2
IPC-MPEG2000	2.5	2.5	5.0	5.0	1.2	89.8
IPC-MPEG5000	1.0	1.0	5.0	5.0	0.5	89.5

MPEG: Methoxy polyethylene glycol, TEA: Triethylamine, THF: Tetrahydrofuran, $CHCl_3$: Chloroform and AC: Acyl Chloride.

3. Spectroscopic characterization

The infrared and ^{1}H, ^{13}C and Dept 135° NMR spectra obtained for each PCMs synthesized are shown below.

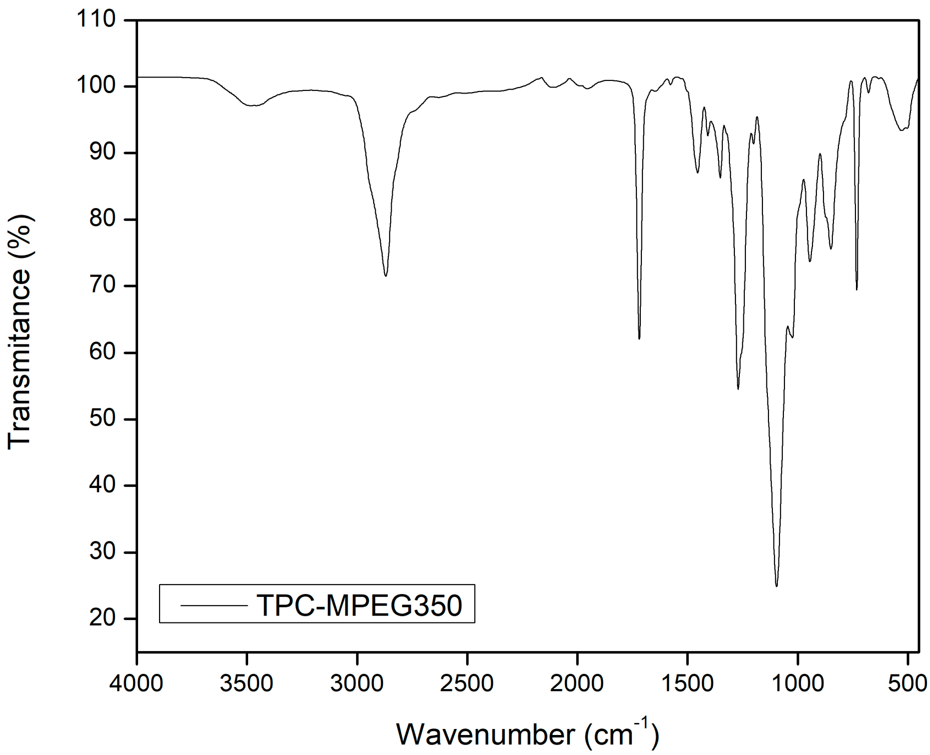
Transmitance (%)
Wavenumber (cm^{-1})
TPC-MPEG350
110
100
90
80
70
60
50
40
30
20
4000
3500
3000
2500
2000
1500
1000
500

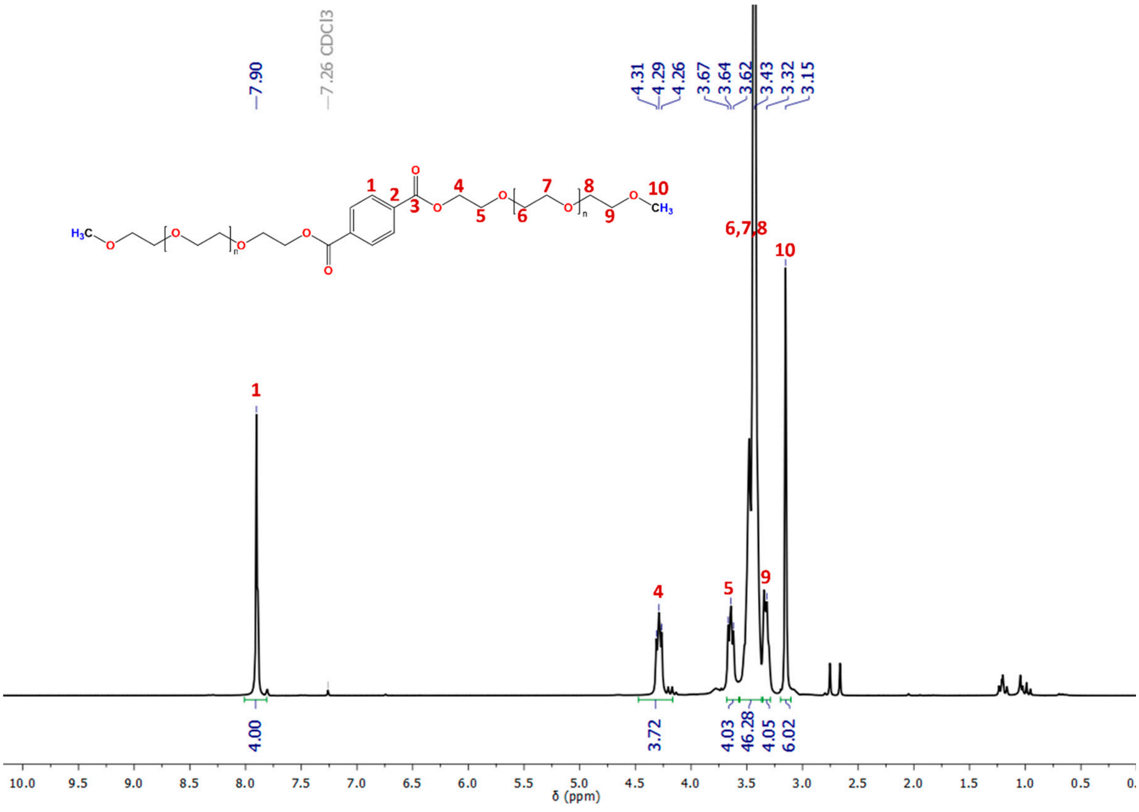
7.90
7.26 CDCl3
4.31
4.29
4.26
3.67
3.64
3.62
3.43
3.32
3.15
6,7,8
10
1
4
5
9
4.00
3.72
4.03
46.28
4.05
6.02
δ (ppm)

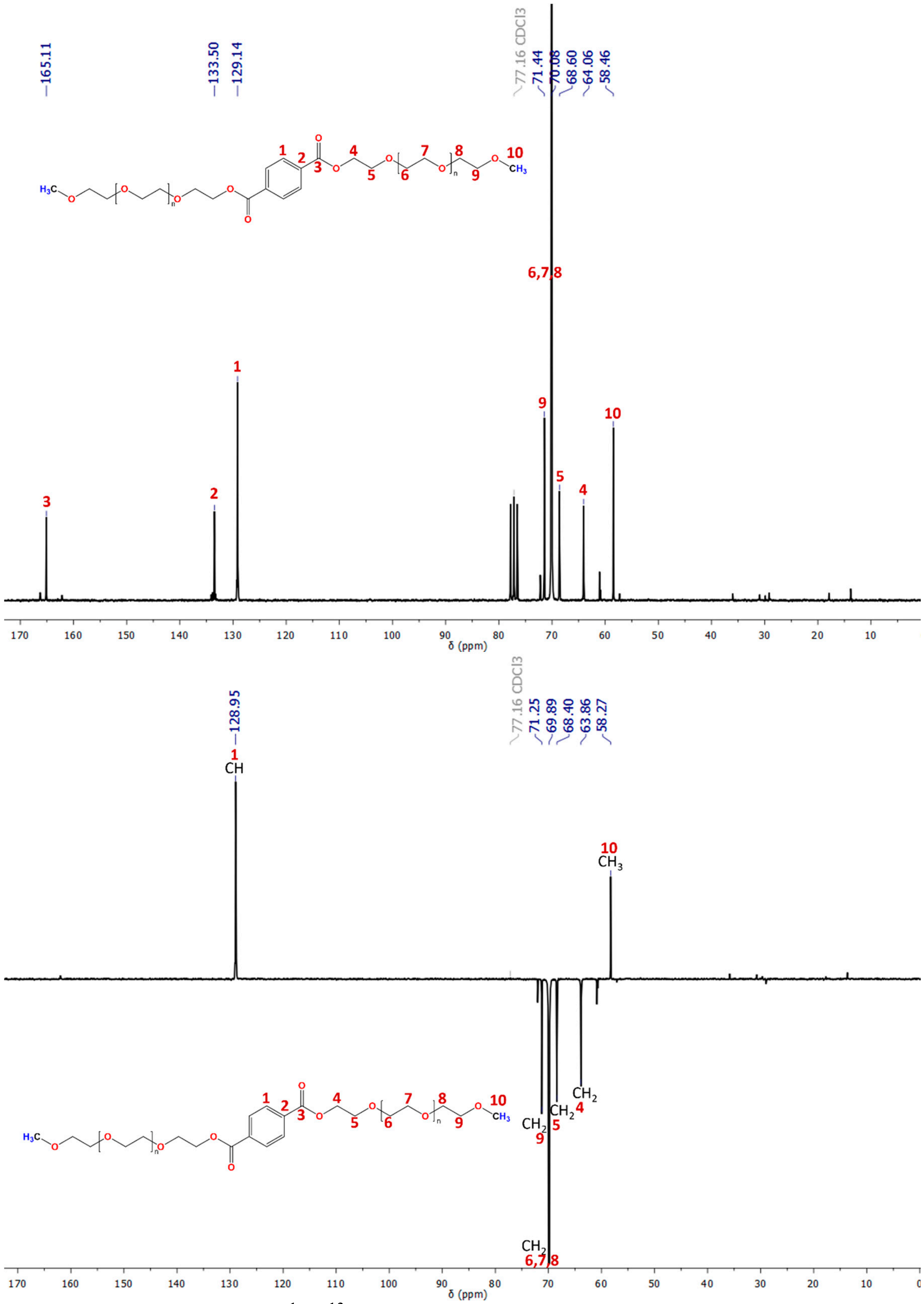

Figure S2. Infrared and ^{1}H, ^{13}C and DEPT 135° NMR spectra of TPC-MPEG350.

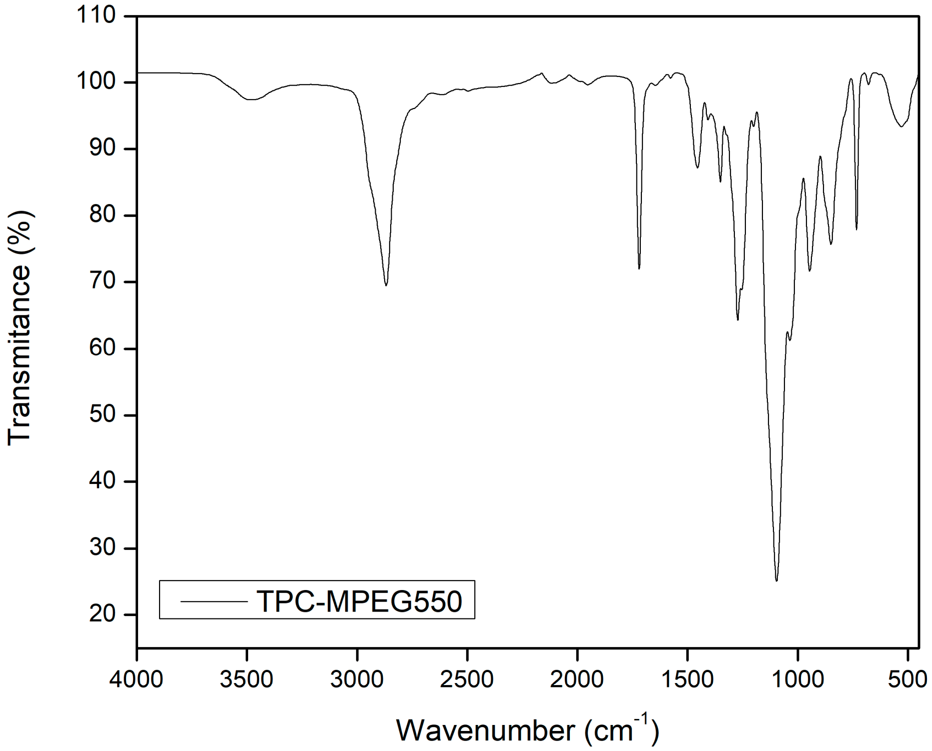

Transmitance (%)
Wavenumber (cm-1)
TPC-MPEG550
110
100
90
80
70
60
50
40
30
20
4000
3500
3000
2500
2000
1500
1000
500

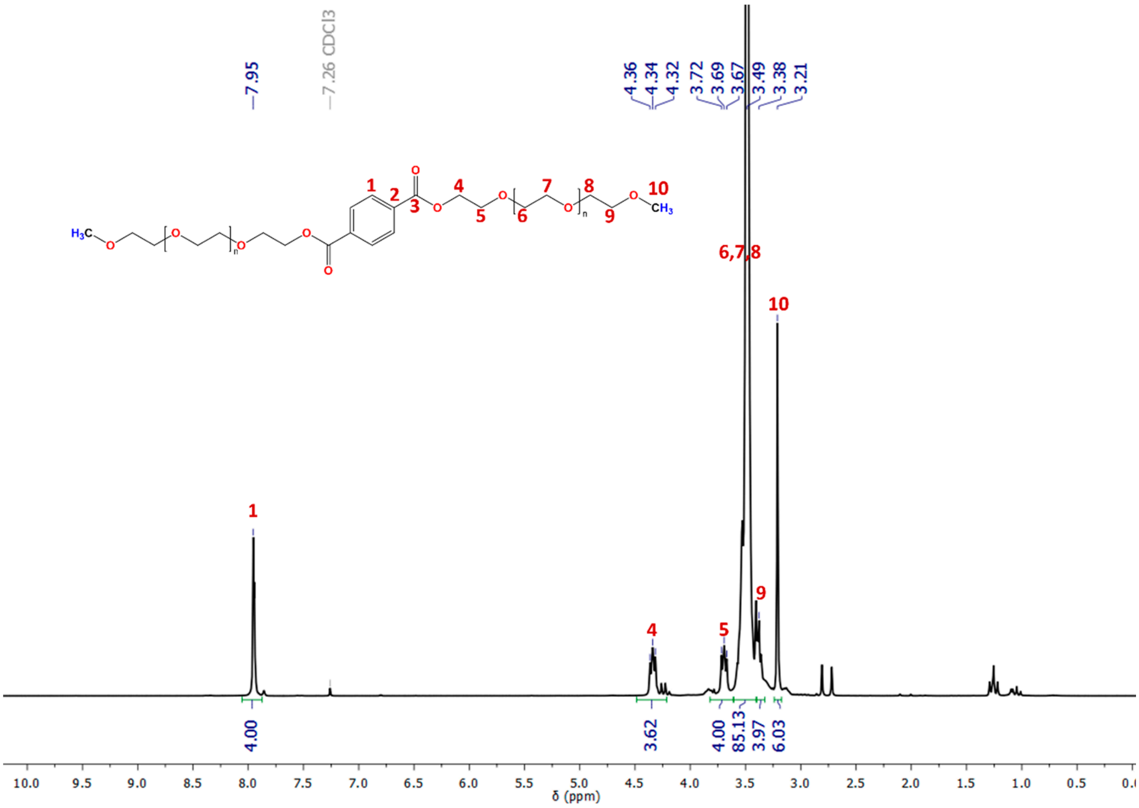

7.95
7.26 CDCl3
4.36
4.34
4.32
3.72
3.69
3.67
3.49
3.38
3.21
6,7,8
10
1
4
5
9
4.00
3.62
4.00
85.13
3.97
6.03
δ (ppm)

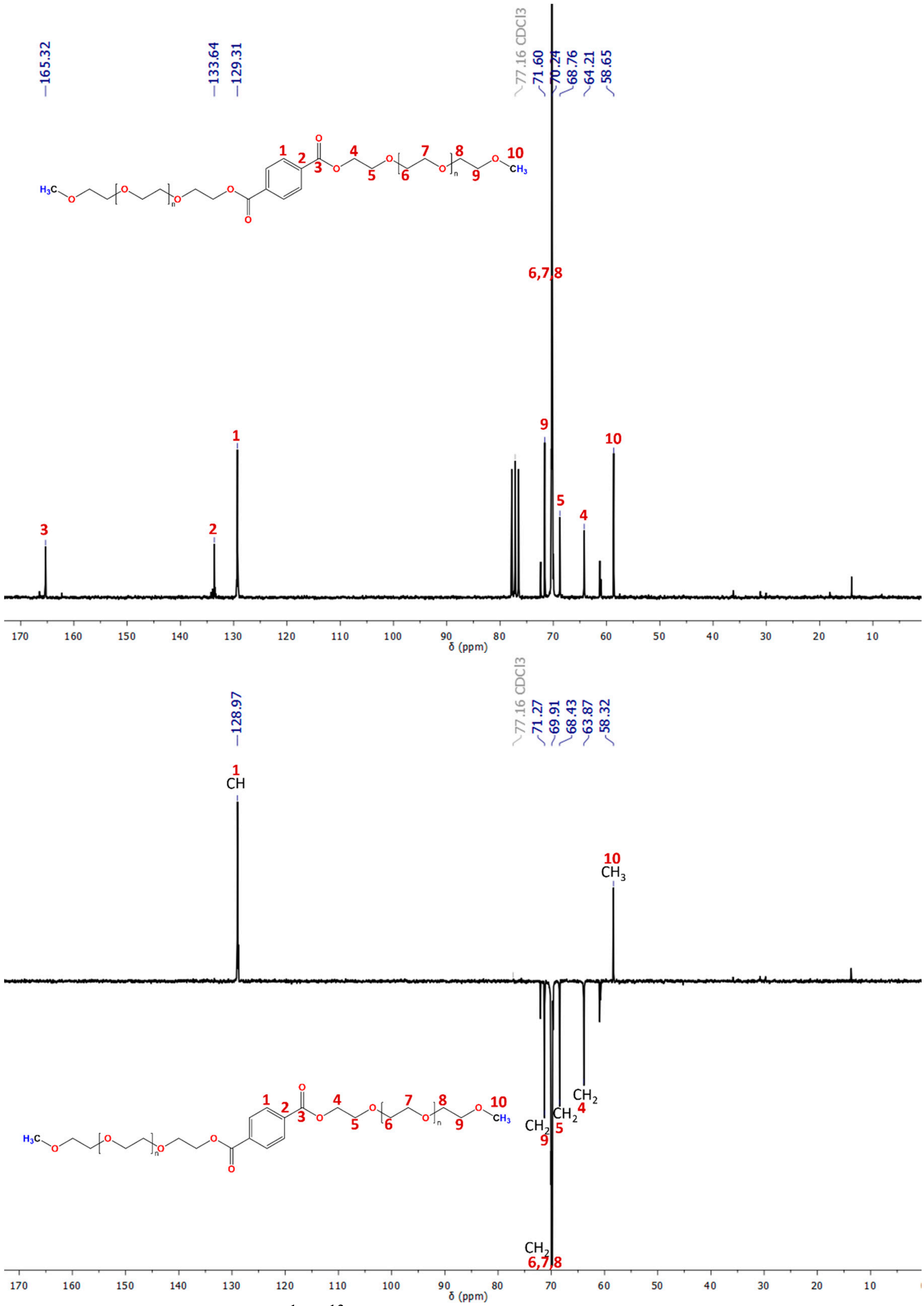

Figure S3. Infrared and ^{1}H, ^{13}C and DEPT 135° NMR spectra of TPC-MPEG550.

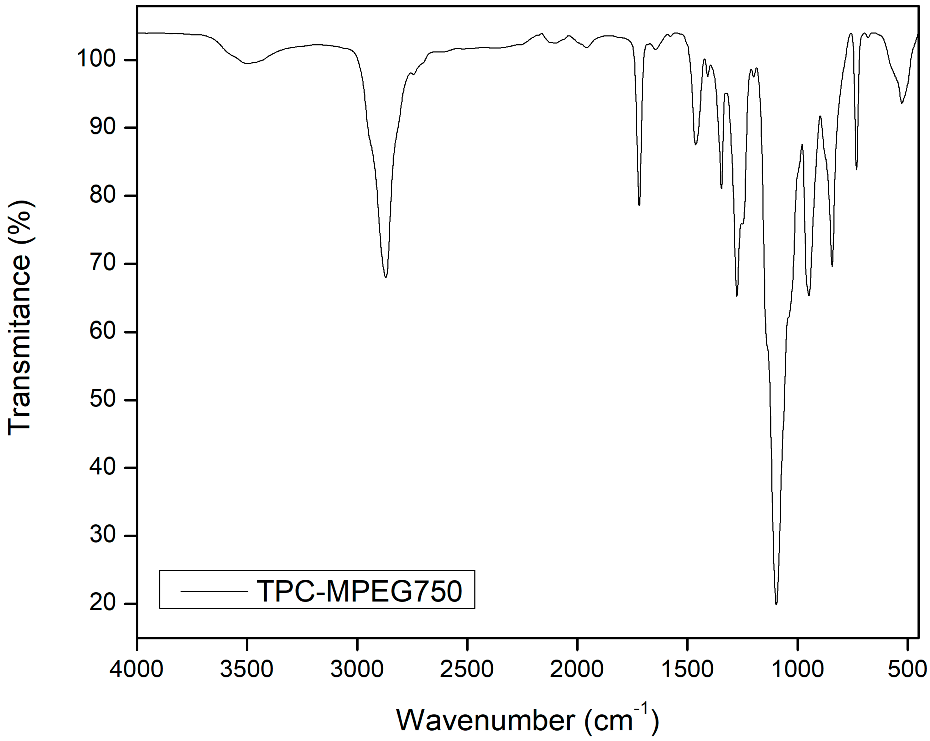
Transmitance (%)
Wavenumber (cm-1)
TPC-MPEG750
100
90
80
70
60
50
40
30
20
4000
3500
3000
2500
2000
1500
1000
500

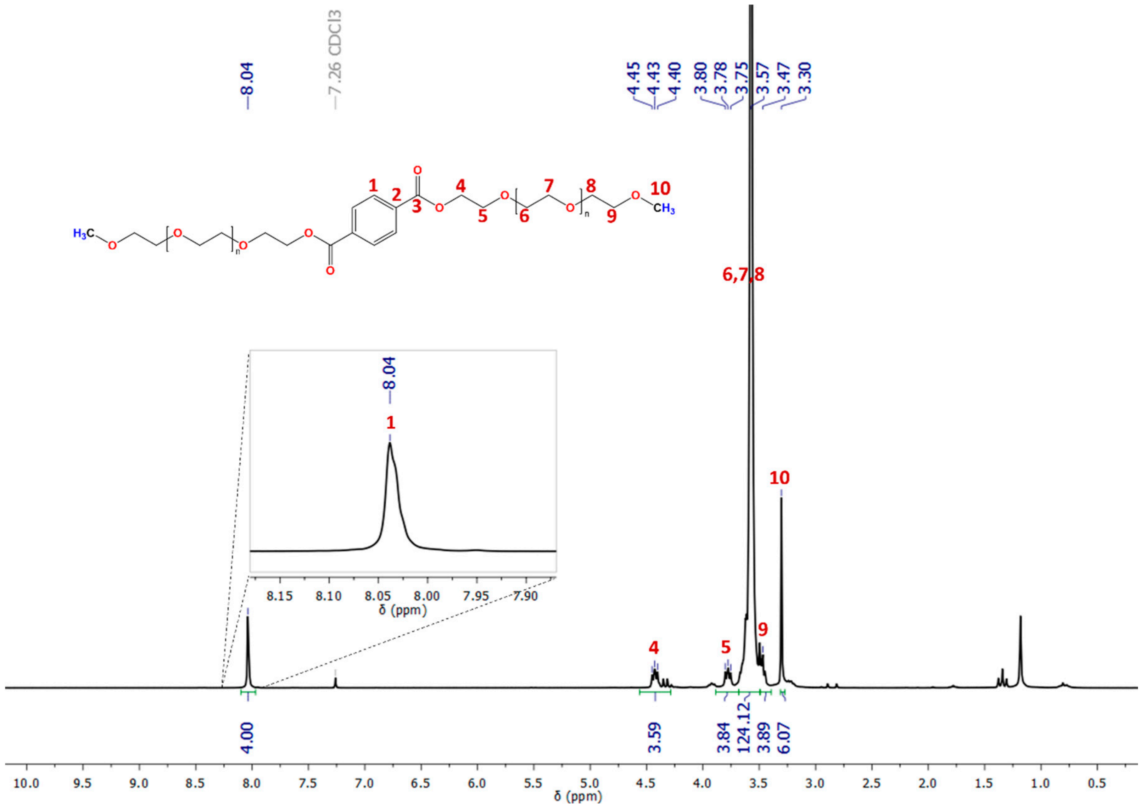
8.04
7.26 CDCl3
4.45
4.43
4.40
3.80
3.78
3.75
3.57
3.47
3.30
6,7,8
1
4
5
9
10
4.00
3.59
3.84
124.12
3.89
6.07
δ (ppm)
8.15
8.10
8.05
8.00
7.95
7.90
10.0
9.5
9.0
8.5
8.0
7.5
7.0
6.5
6.0
5.5
5.0
4.5
4.0
3.5
3.0
2.5
2.0
1.5
1.0
0.5

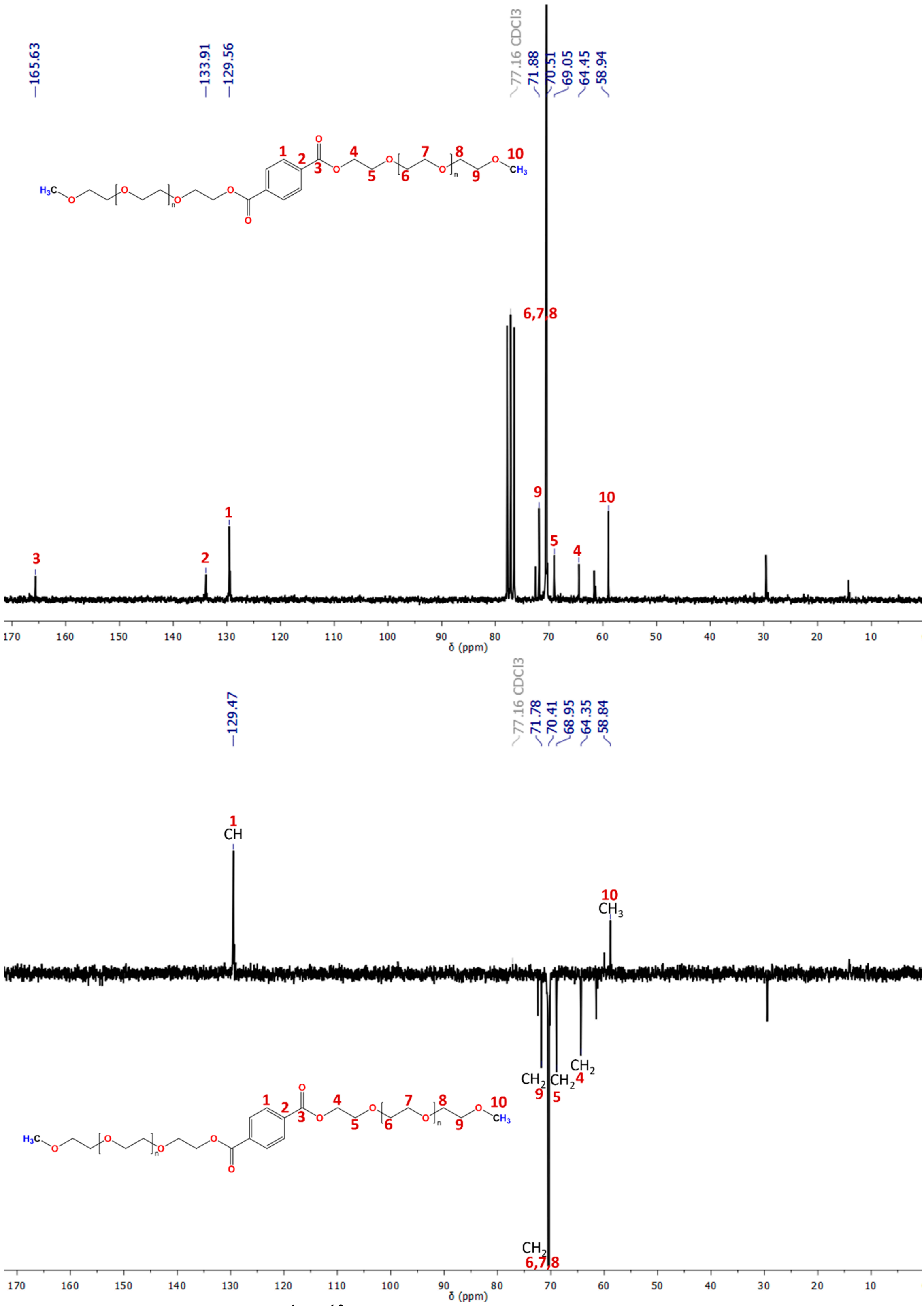

Figure S4. Infrared and ^{1}H, ^{13}C and DEPT 135° NMR spectra of TPC-MPEG750.

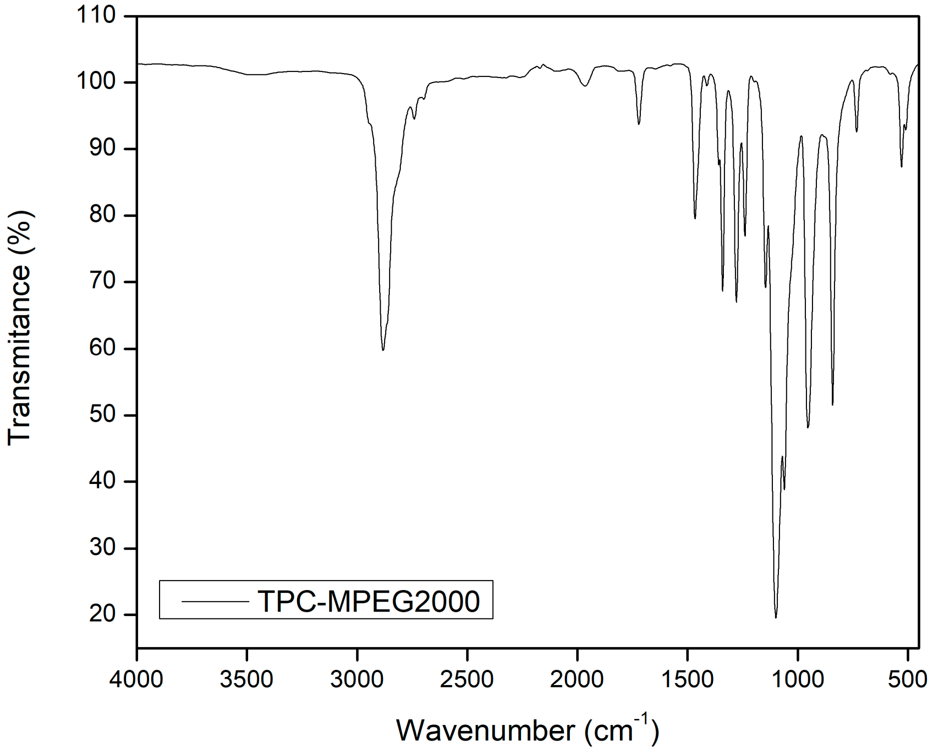

Transmitance (%)
Wavenumber (cm-1)
TPC-MPEG2000
110
100
90
80
70
60
50
40
30
20
4000
3500
3000
2500
2000
1500
1000
500

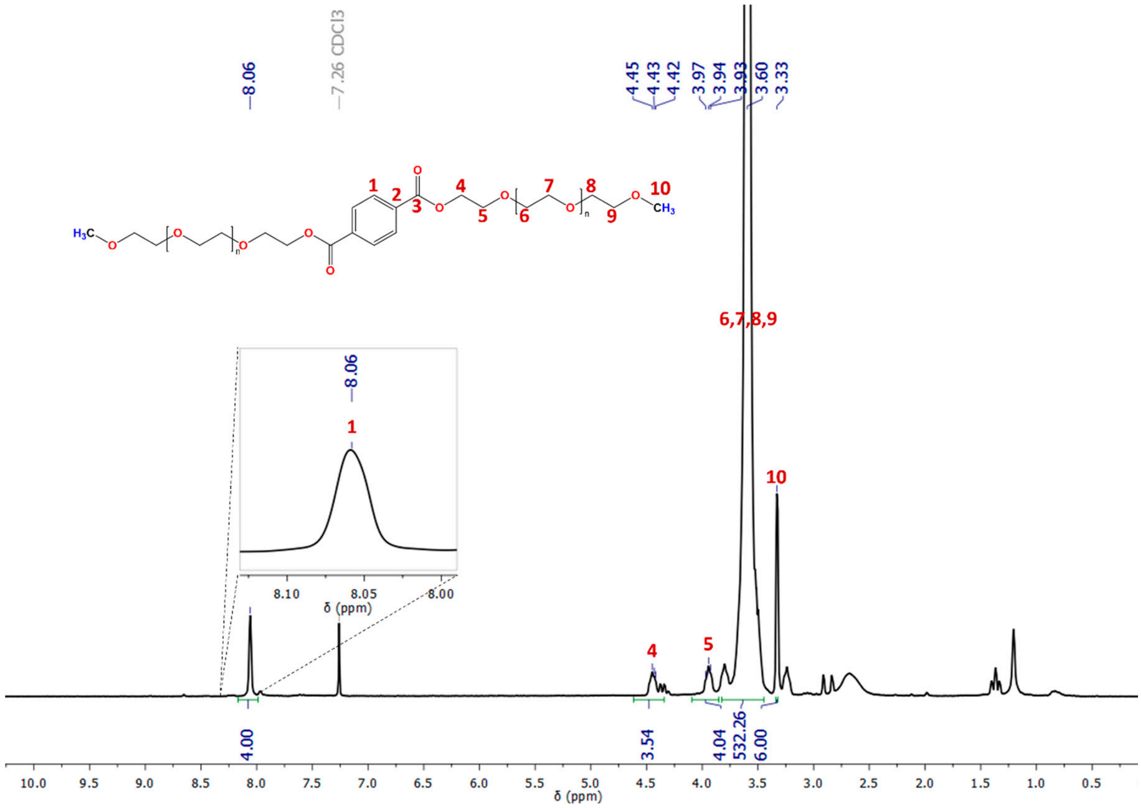

8.06
7.26 CDCl3
4.45
4.43
4.42
3.97
3.94
3.93
3.60
3.33
6,7,8,9
10
1
4
5
4.00
3.54
4.04
532.26
6.00
δ (ppm)

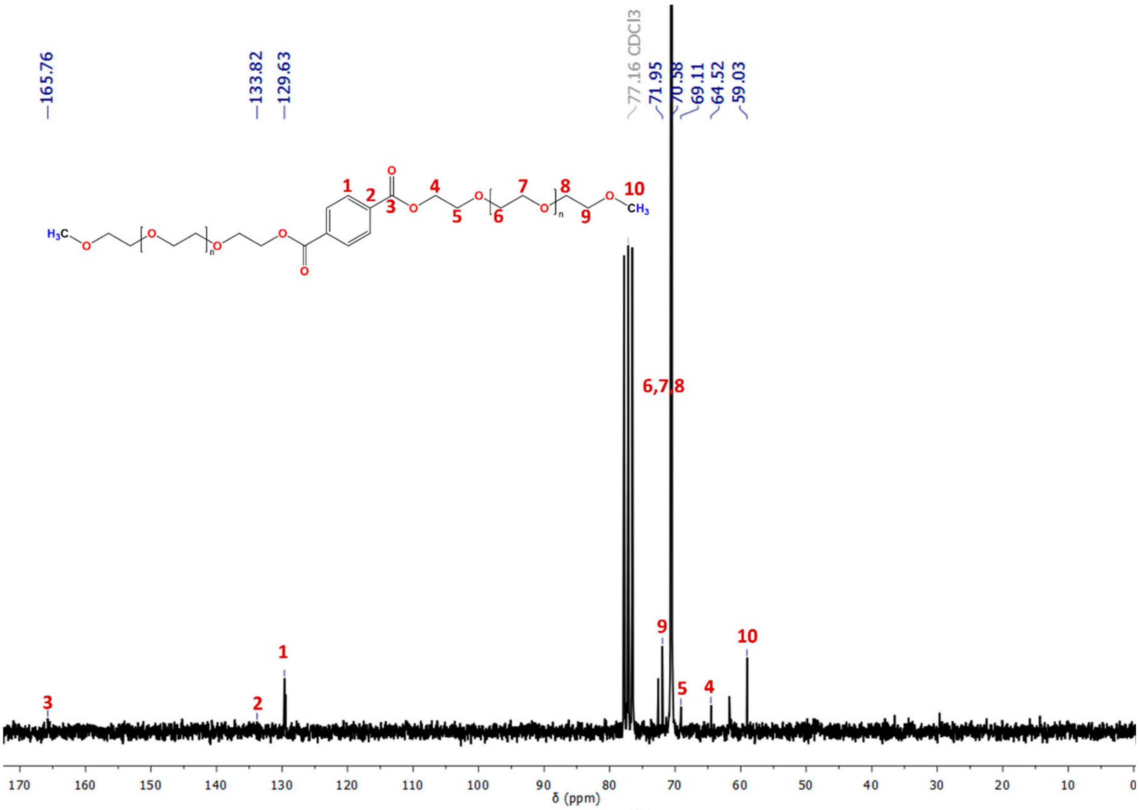

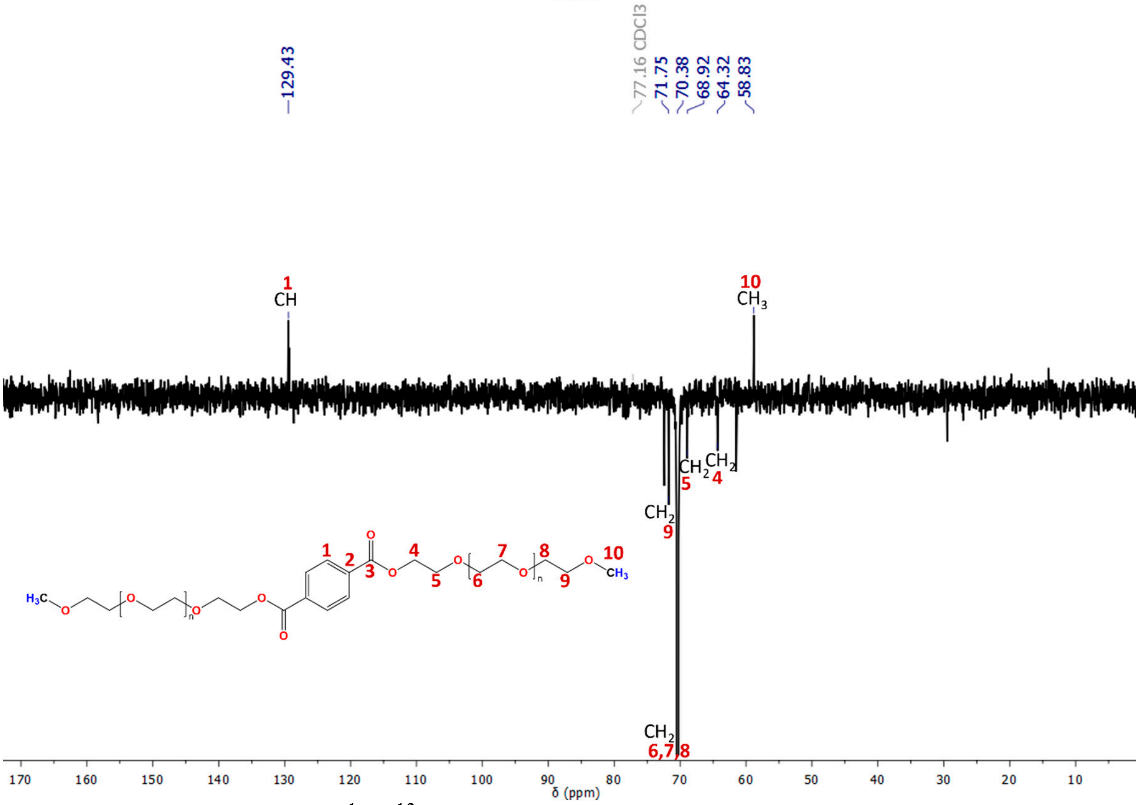

Figure S5. Infrared and ^{1}H, ^{13}C and DEPT 135° NMR spectra of TPC-MPEG2000.

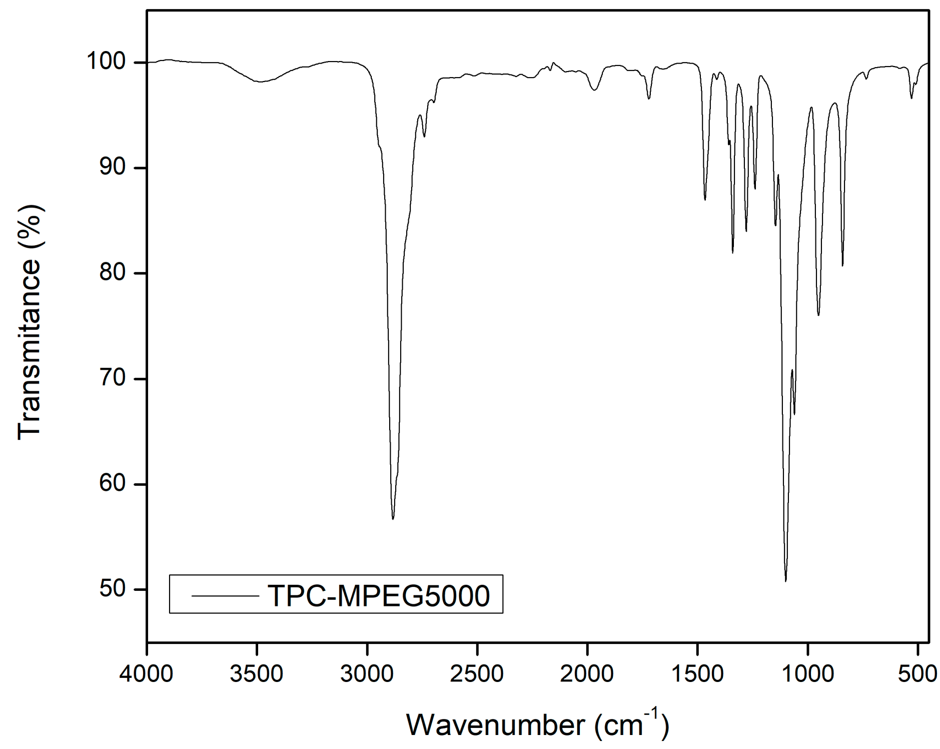
Transmitance (%)
Wavenumber (cm-1)
TPC-MPEG5000

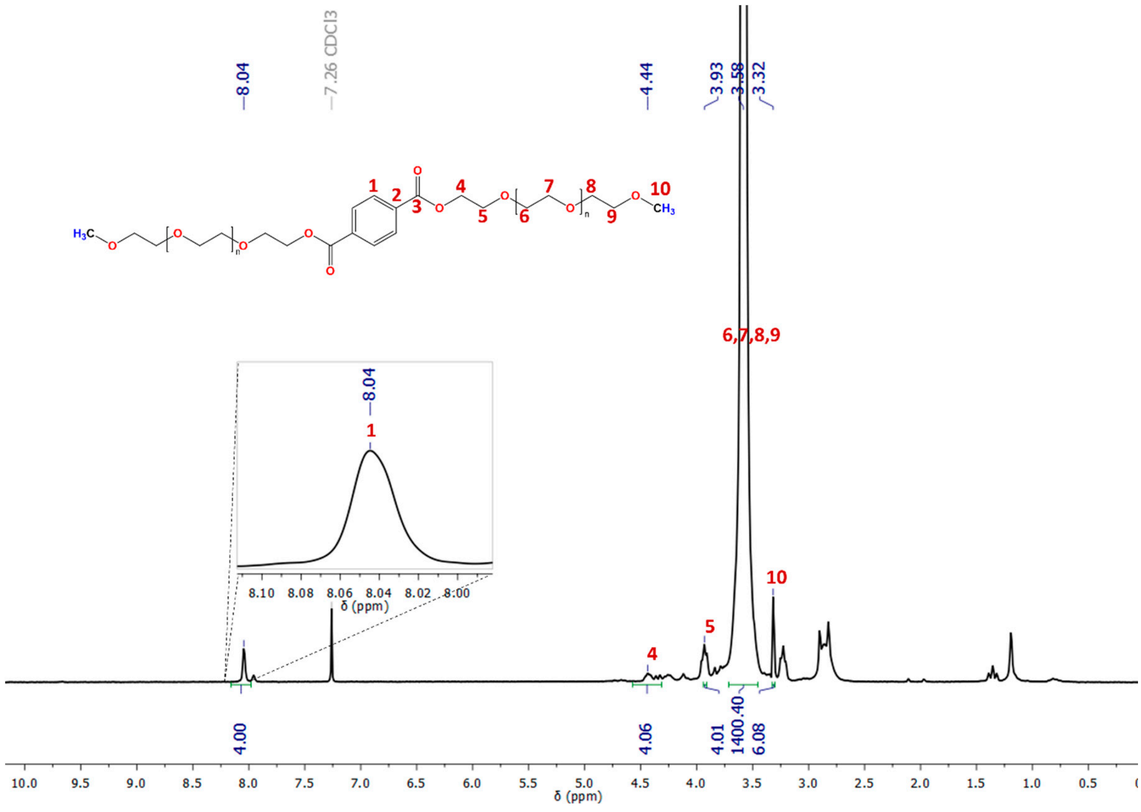
8.04
7.26 CDCl3
4.44
3.93
3.58
3.32
6,7,8,9
10
5
4
1
4.00
4.06
4.01
1400.40
6.08
δ (ppm)

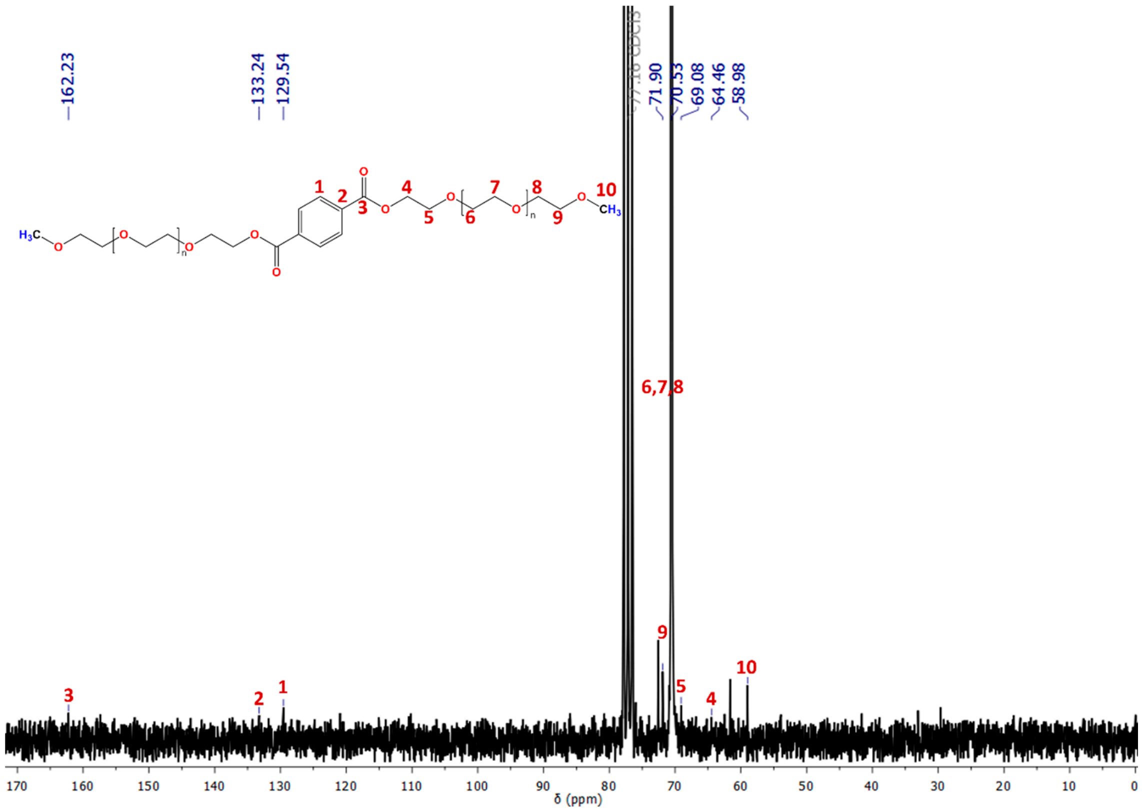

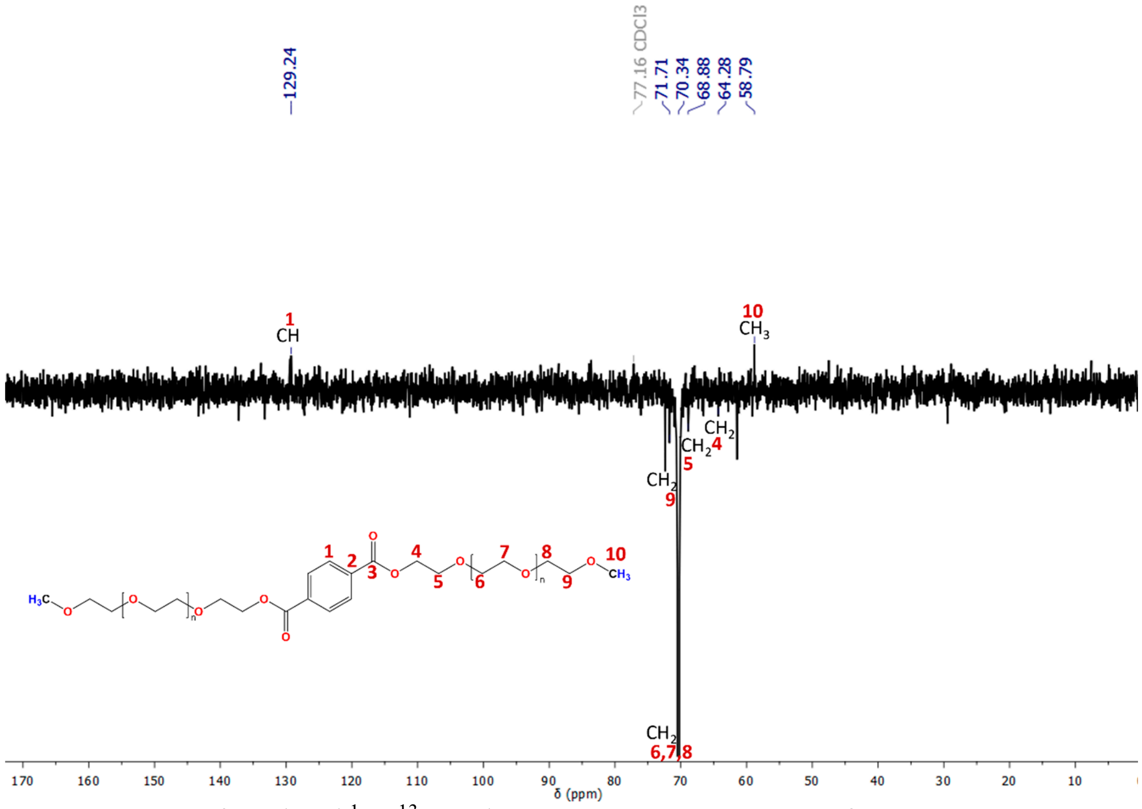

Figure S6. Infrared and ^{1}H, ^{13}C and DEPT 135° NMR spectra of TPC-MPEG5000.

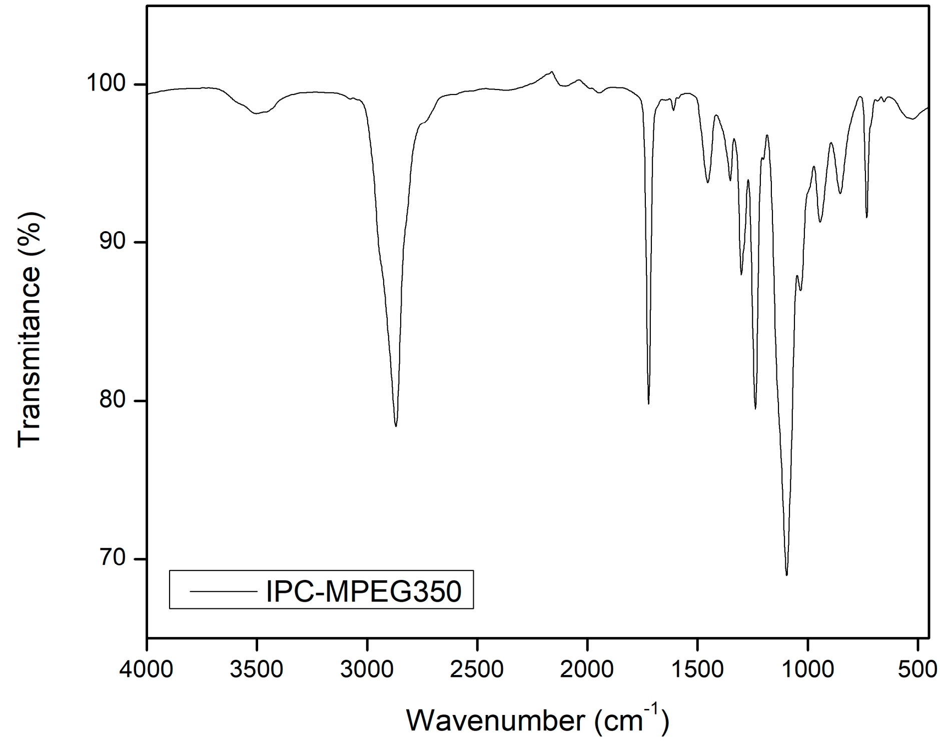
Transmitance (%)
100
90
80
70
IPC-MPEG350
4000
3500
3000
2500
2000
1500
1000
500
Wavenumber (cm-1)

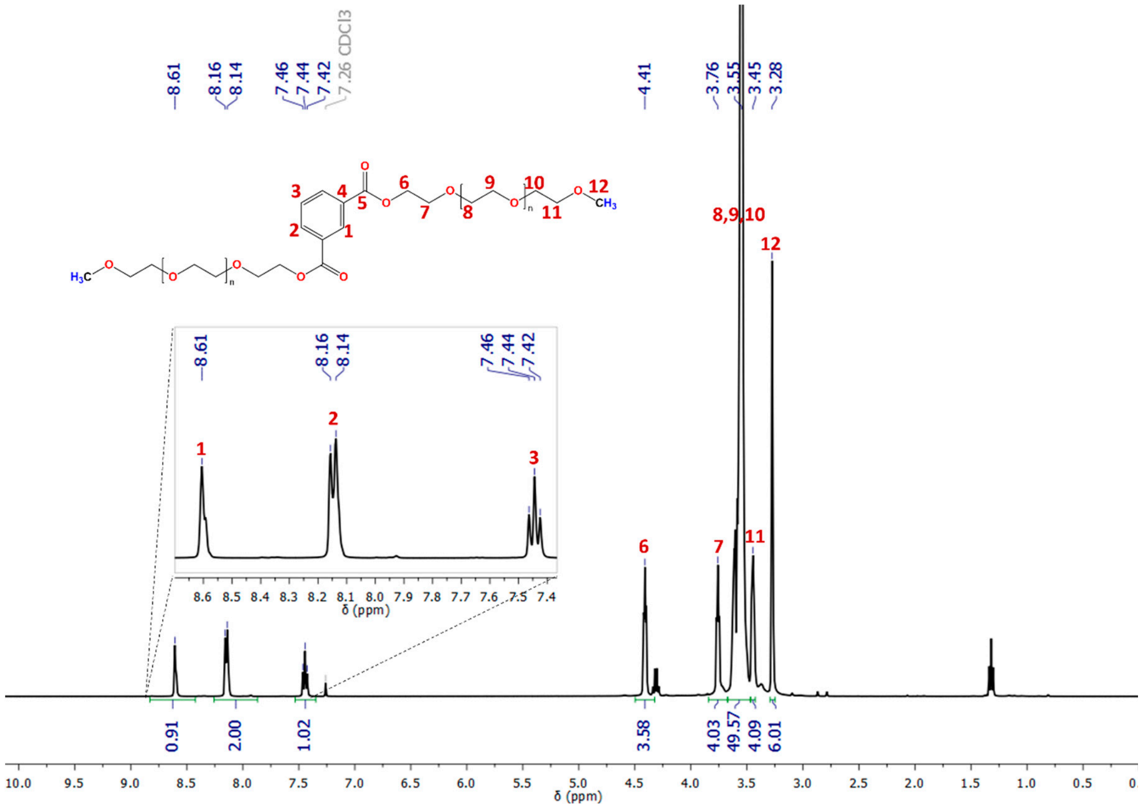
8.61
8.16
8.14
7.46
7.44
7.42
7.26 CDCl3
4.41
3.76
3.55
3.45
3.28
1
2
3
4
5
6
7
8
9
10
11
12
8,9,10
0.91
2.00
1.02
3.58
4.03
49.57
4.09
6.01
δ (ppm)

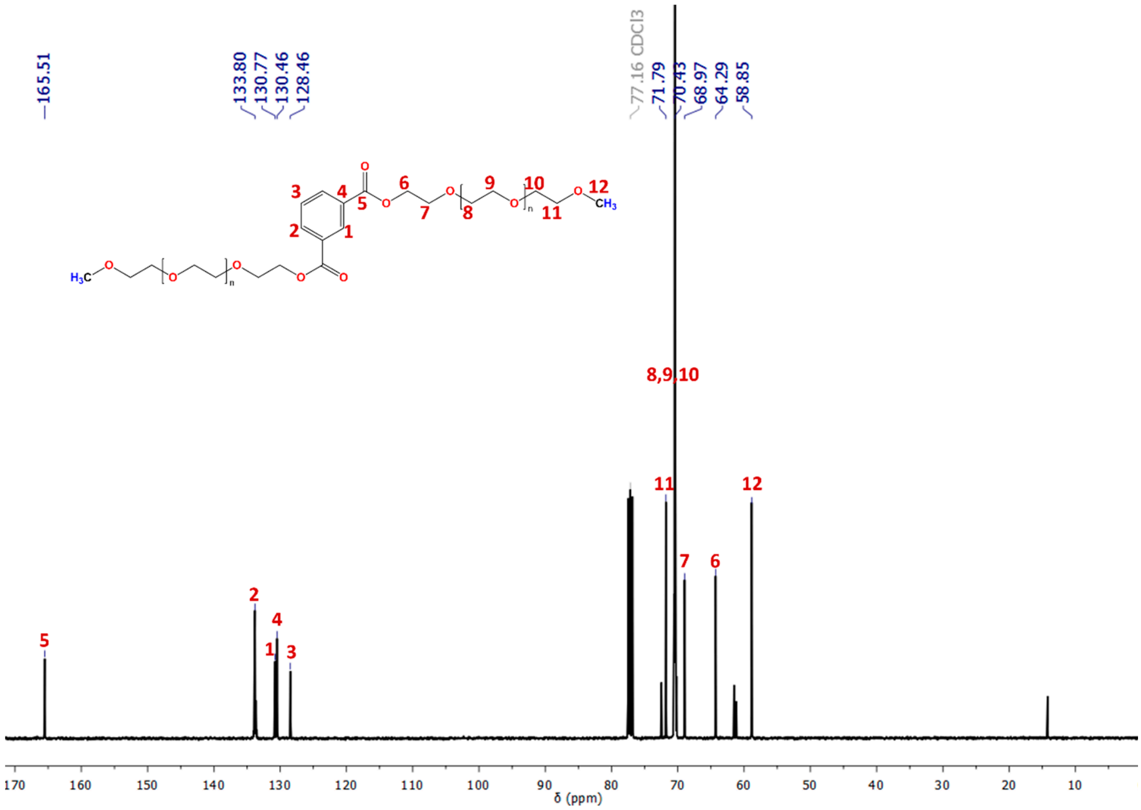

165.51
133.80
130.77
130.46
128.46
77.16 CDCl3
71.79
70.43
68.97
64.29
58.85
8,9,10
11
12
7
6
5
2
4
1
3
δ (ppm)

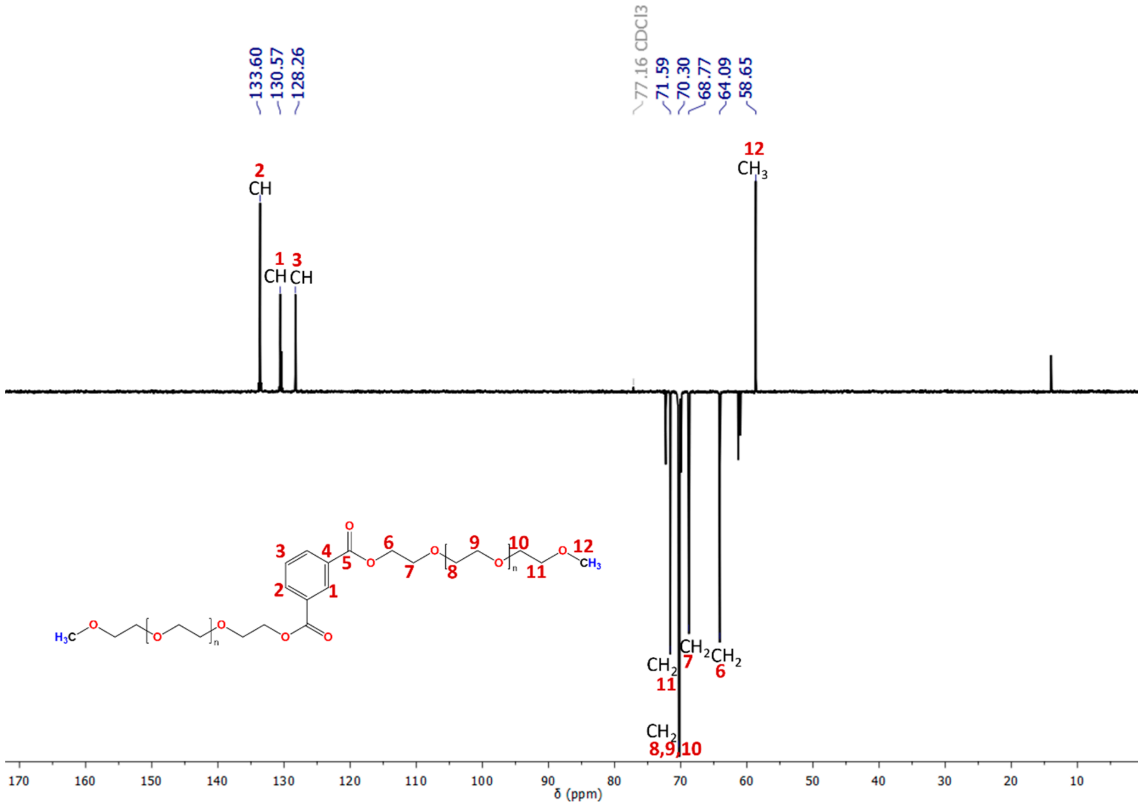

133.60
130.57
128.26
77.16 CDCl3
71.59
70.30
68.77
64.09
58.65
12
CH3
2
CH
1
CH
3
CH
CH2
11
CH2
7
CH2
6
CH2
8,9,10
δ (ppm)

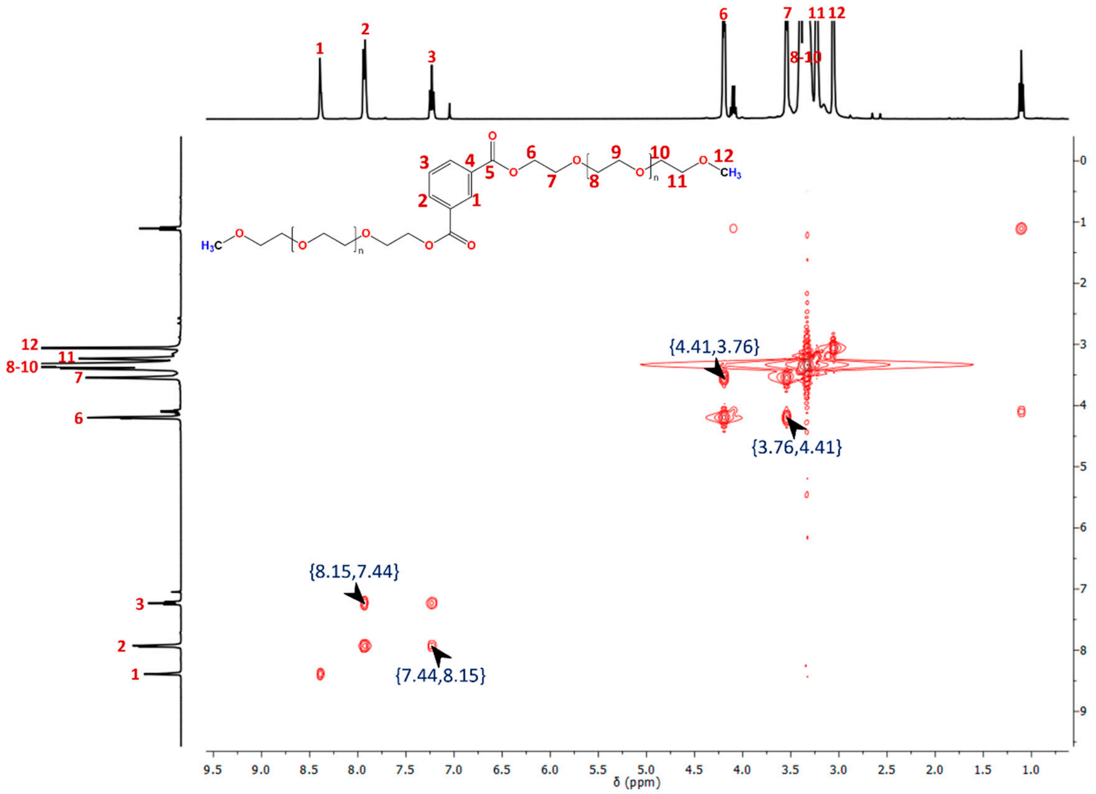

1
2
3
6
7
8-10
11
12
4
5
9
10
{4.41,3.76}
{3.76,4.41}
{8.15,7.44}
{7.44,8.15}
δ (ppm)

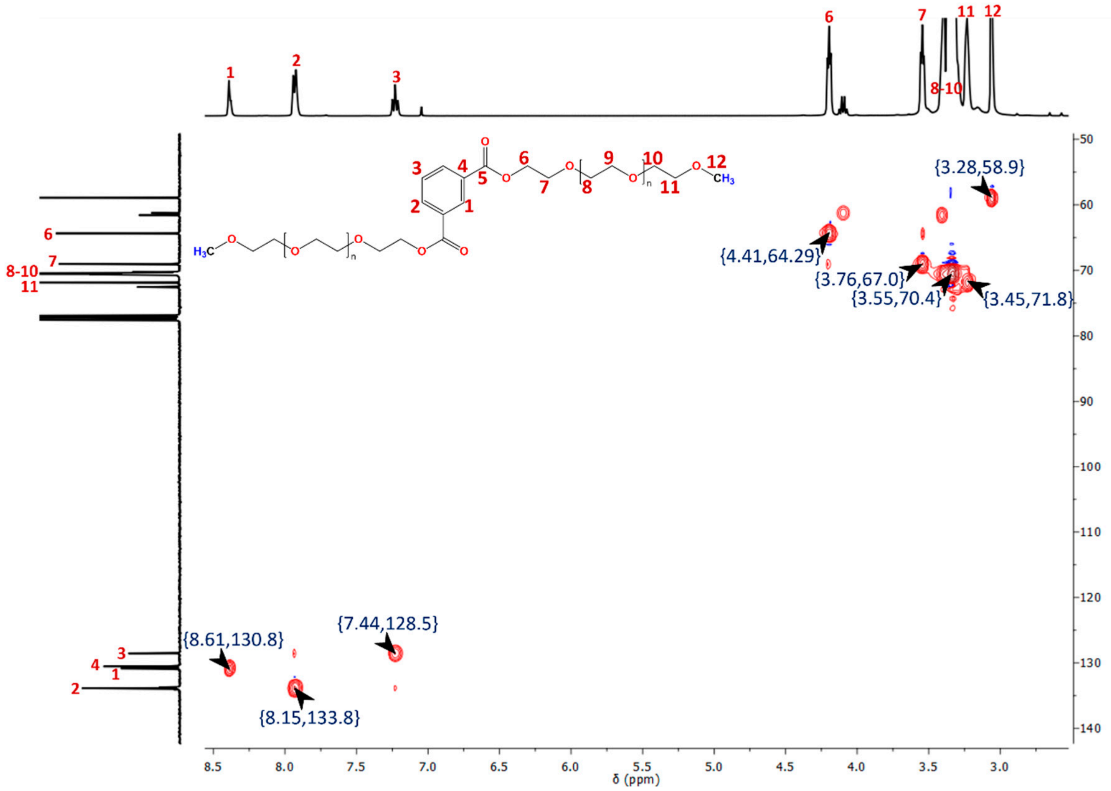

1
2
3
4
5
6
7
8-10
11
12
{3.28,58.9}
{4.41,64.29}
{3.76,67.0}
{3.55,70.4}
{3.45,71.8}
{8.61,130.8}
{7.44,128.5}
{8.15,133.8}
δ (ppm)

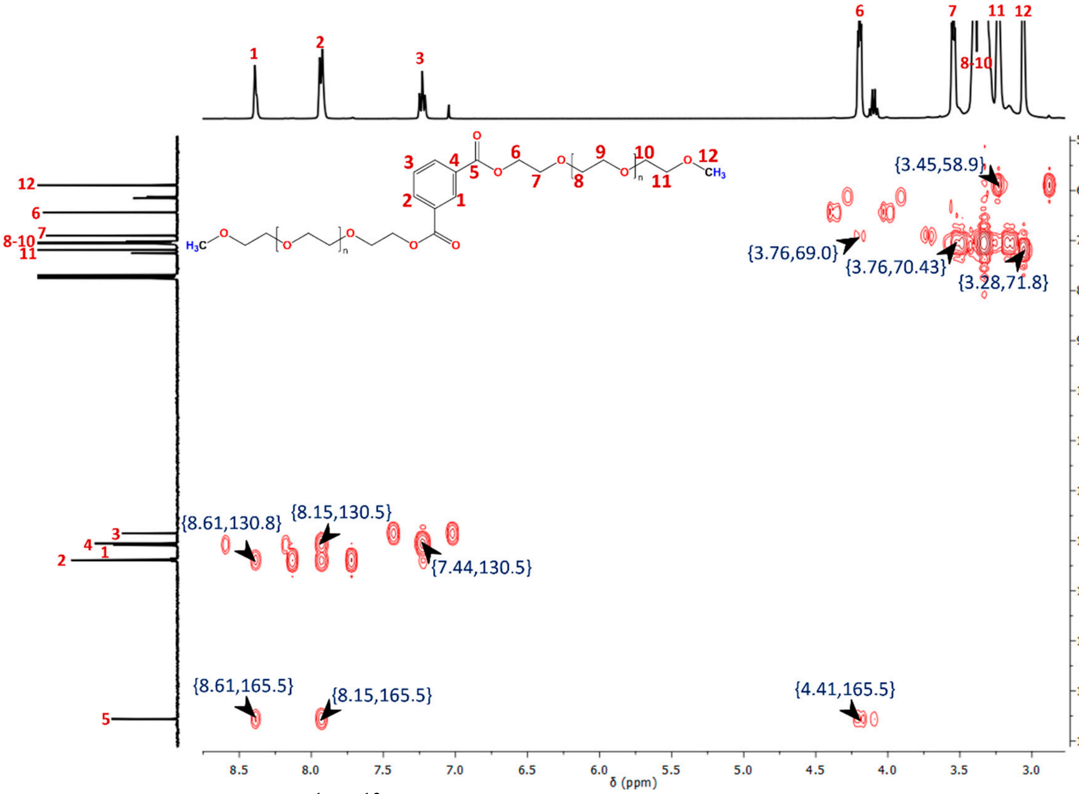

Figure S7. Infrared and ^{1}H, ^{13}C, DEPT 135°, COSY, HMQC and HMBC NMR spectra of IPC-MPEG350.

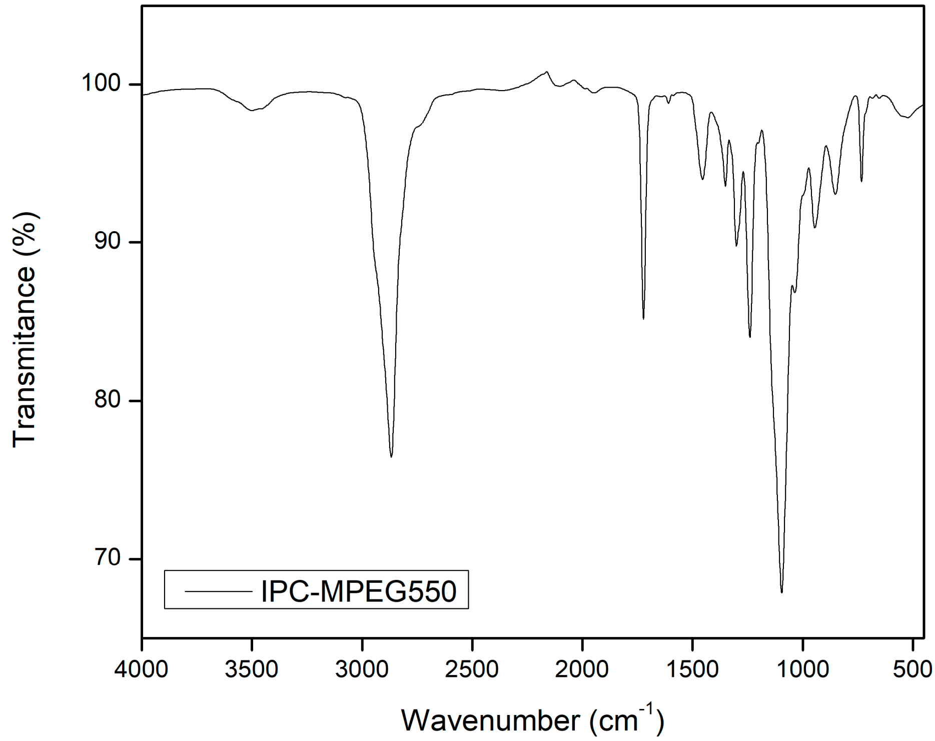
Transmitance (%)
Wavenumber (cm-1)
IPC-MPEG550
100
90
80
70
4000
3500
3000
2500
2000
1500
1000
500

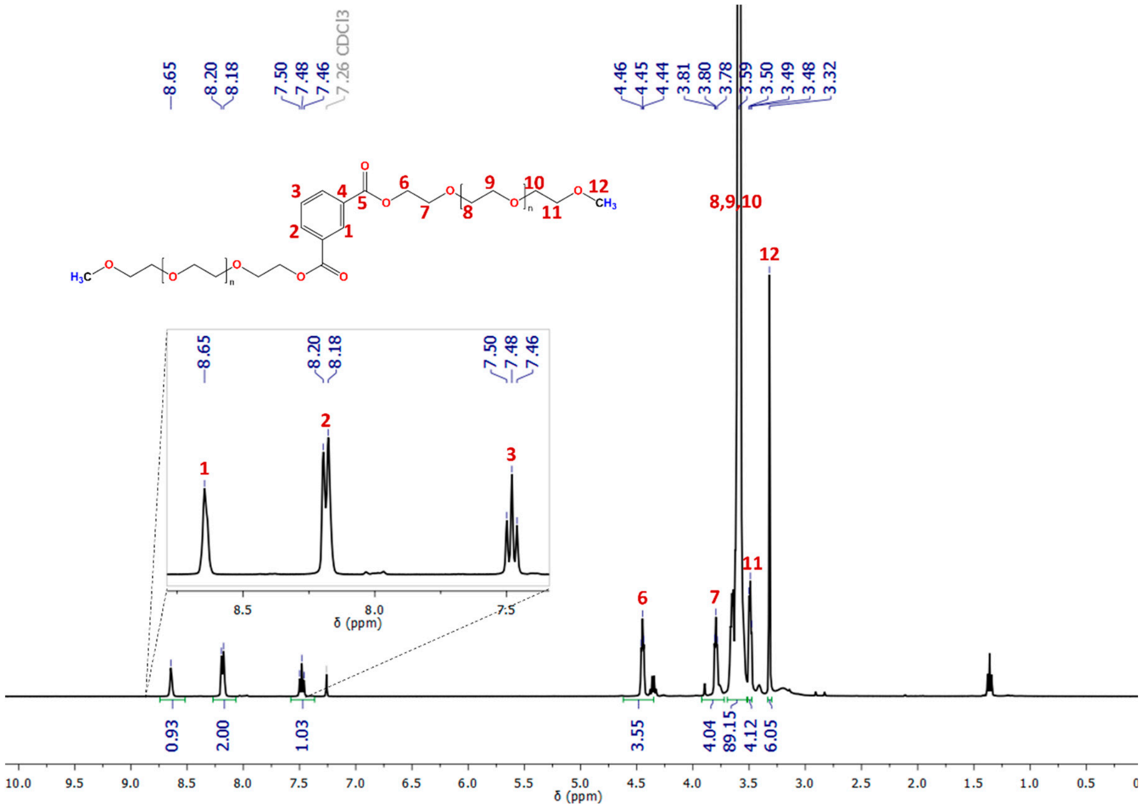
8.65
8.20
8.18
7.50
7.48
7.46
7.26 CDCl3
4.46
4.45
4.44
3.81
3.80
3.78
3.59
3.50
3.49
3.48
3.32
8,9,10
12
11
7
6
1
2
3
4
5
0.93
2.00
1.03
3.55
4.04
89.15
4.12
6.05
δ (ppm)

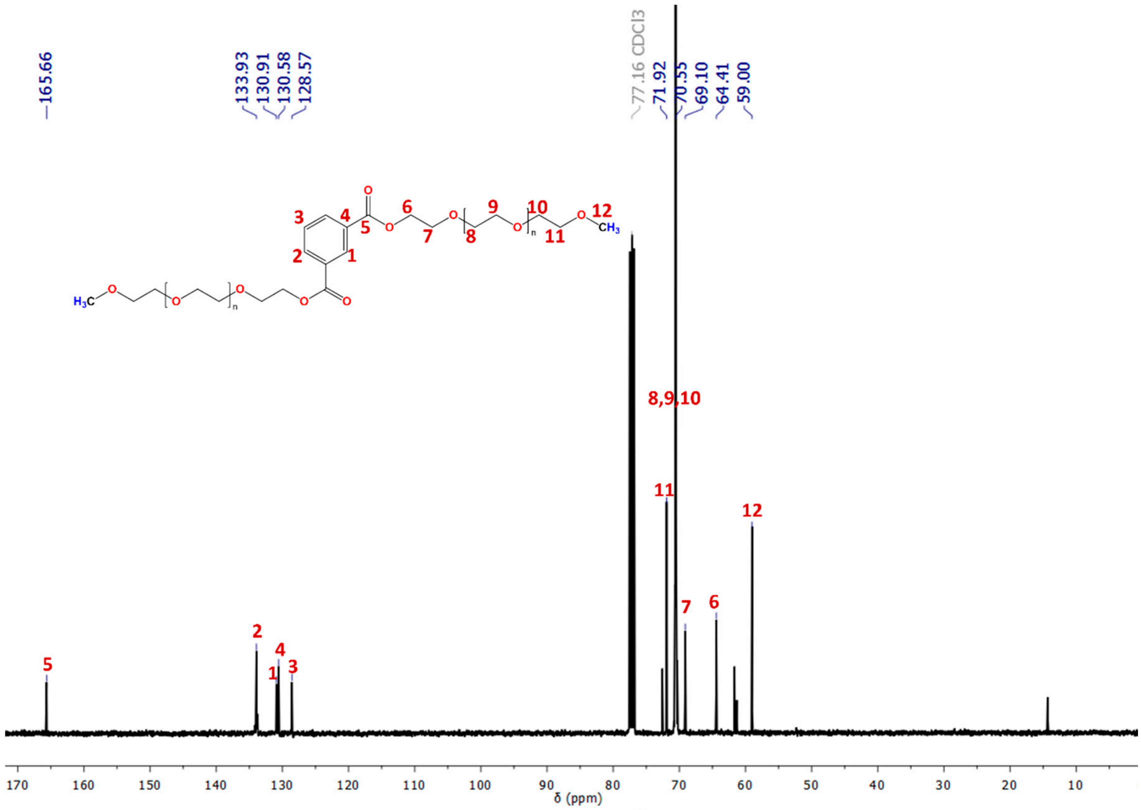

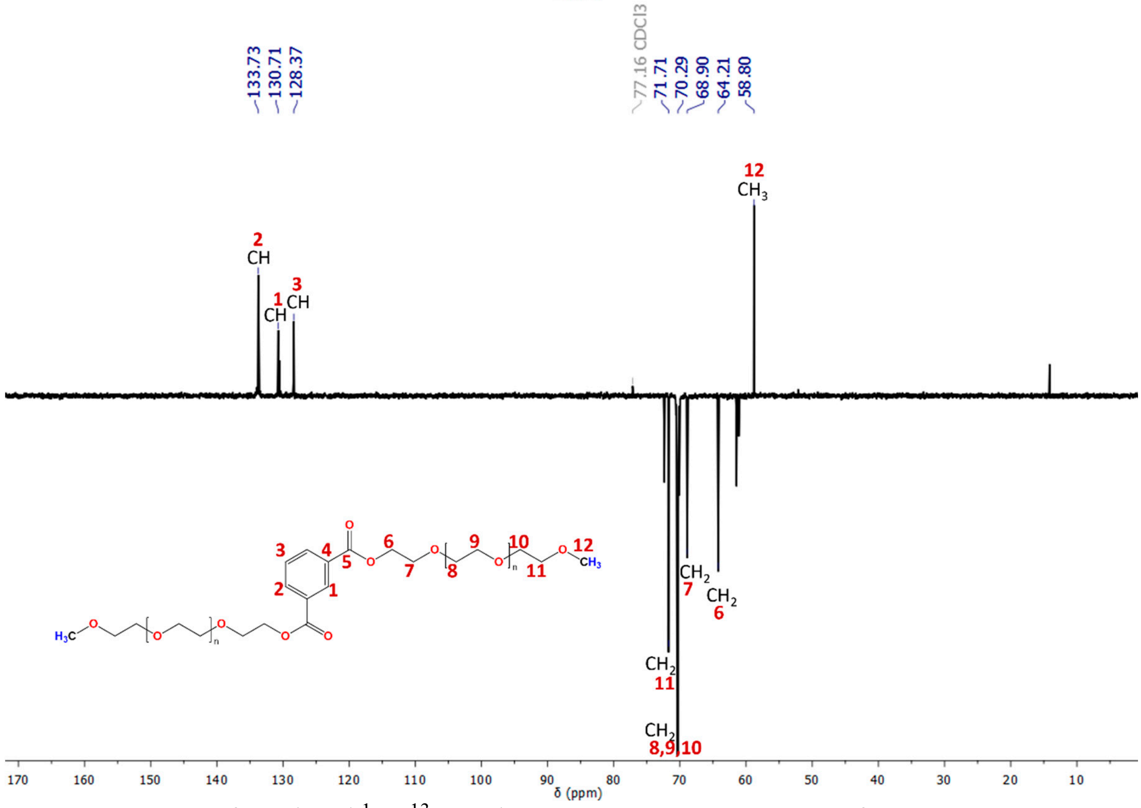

Figure S8. Infrared and ^{1}H, ^{13}C and DEPT 135° NMR spectra of IPC-MPEG550.

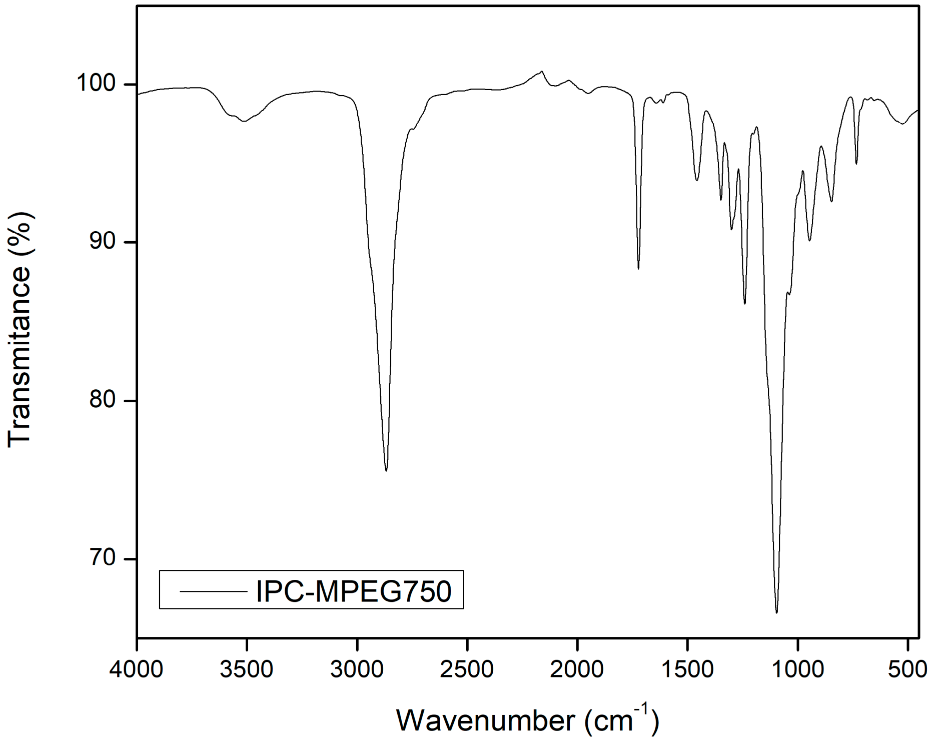

Transmitance (%)
100
90
80
70
IPC-MPEG750
4000
3500
3000
2500
2000
1500
1000
500
Wavenumber (cm-1)

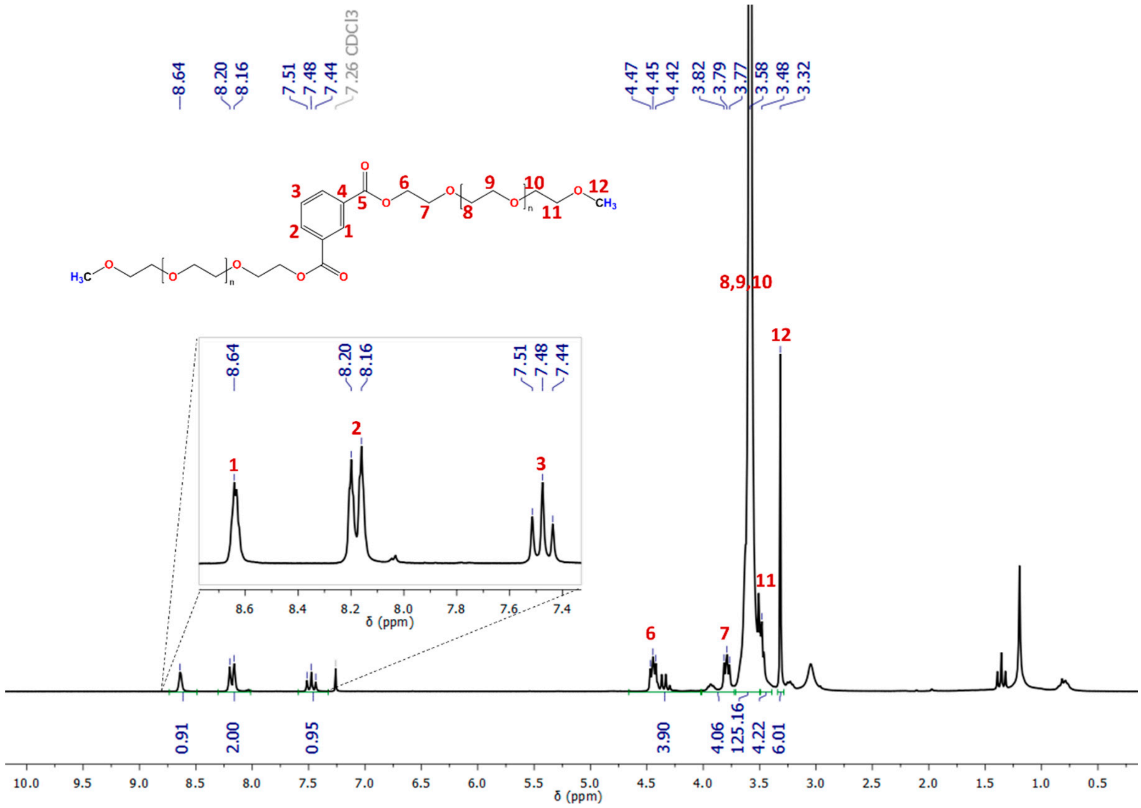

8.64
8.20
8.16
7.51
7.48
7.44
7.26 CDCl3
4.47
4.45
4.42
3.82
3.79
3.77
3.58
3.48
3.32
8,9,10
12
11
6
7
1
2
3
0.91
2.00
0.95
3.90
4.06
125.16
4.22
6.01
δ (ppm)

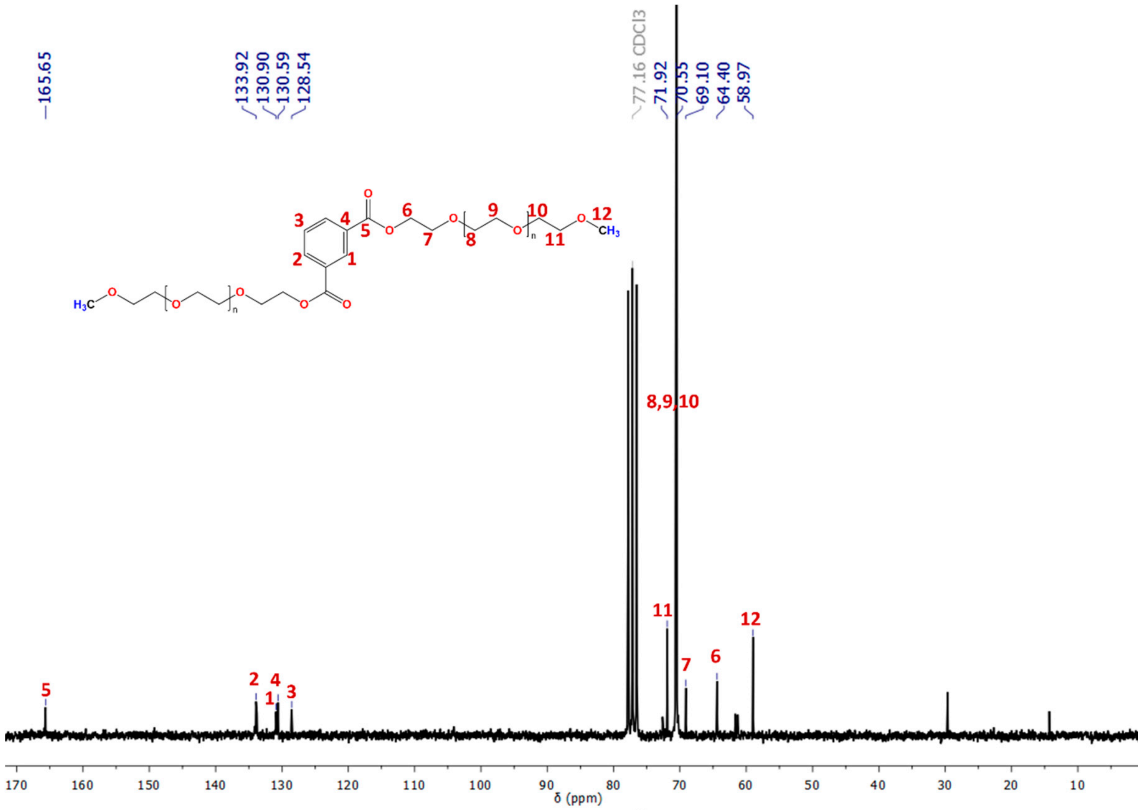

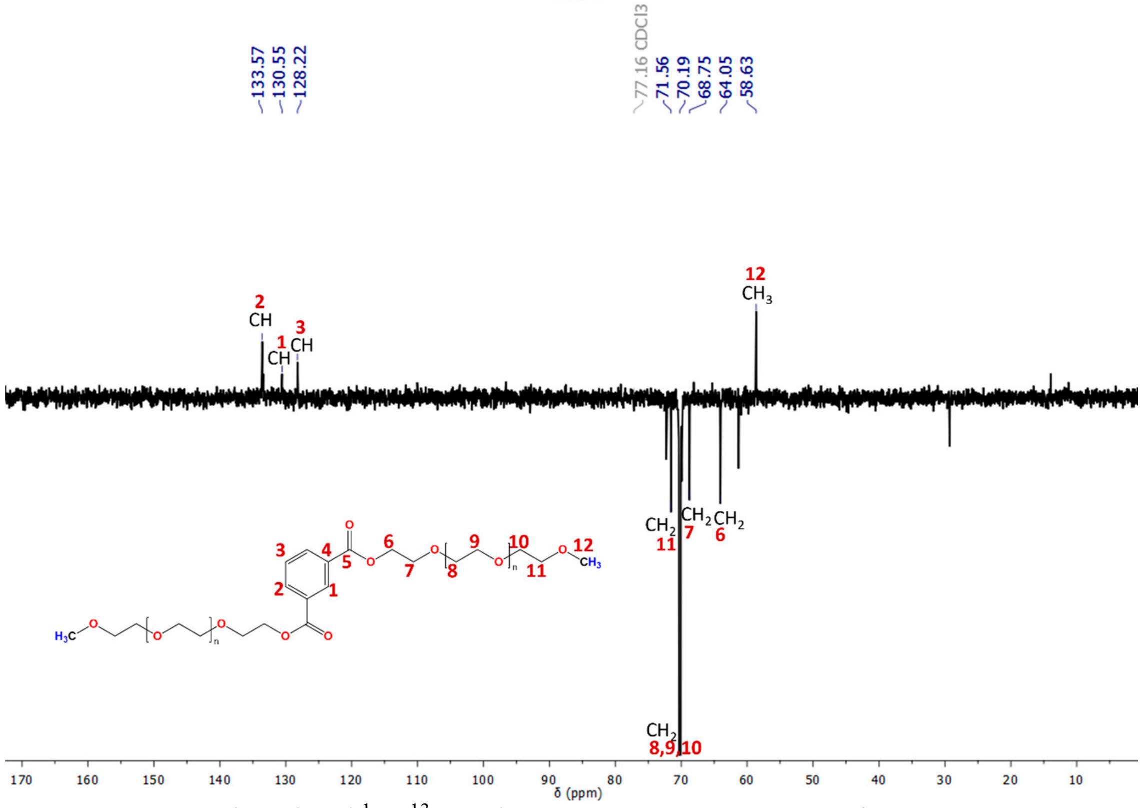

Figure S9. Infrared and ^{1}H, ^{13}C and DEPT 135° NMR spectra of IPC-MPEG750.

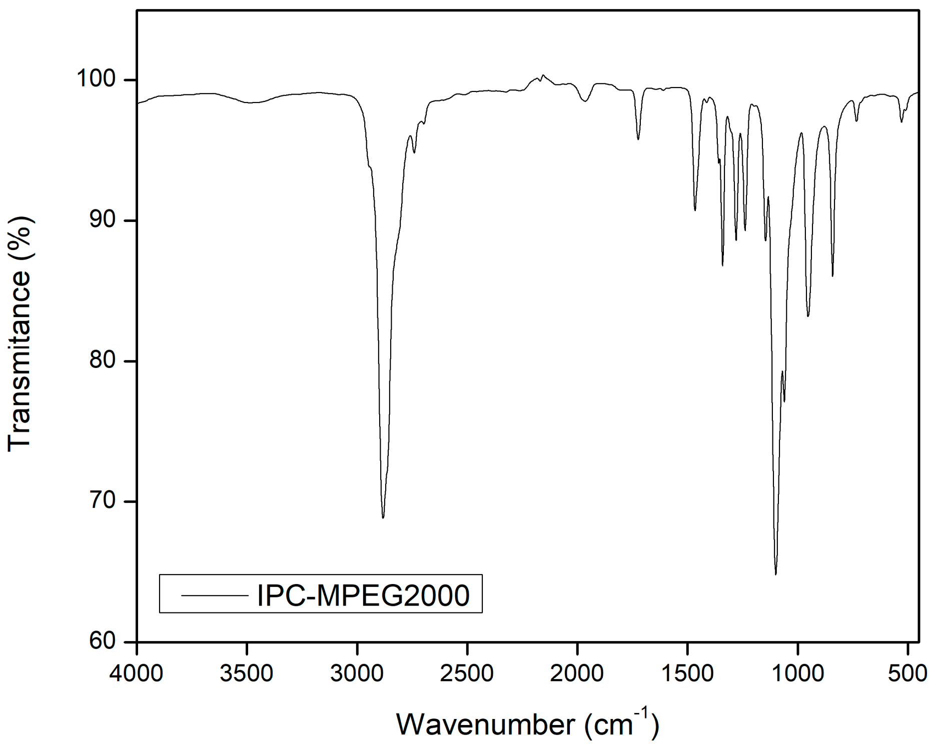

Transmitance (%)
Wavenumber (cm-1)
IPC-MPEG2000

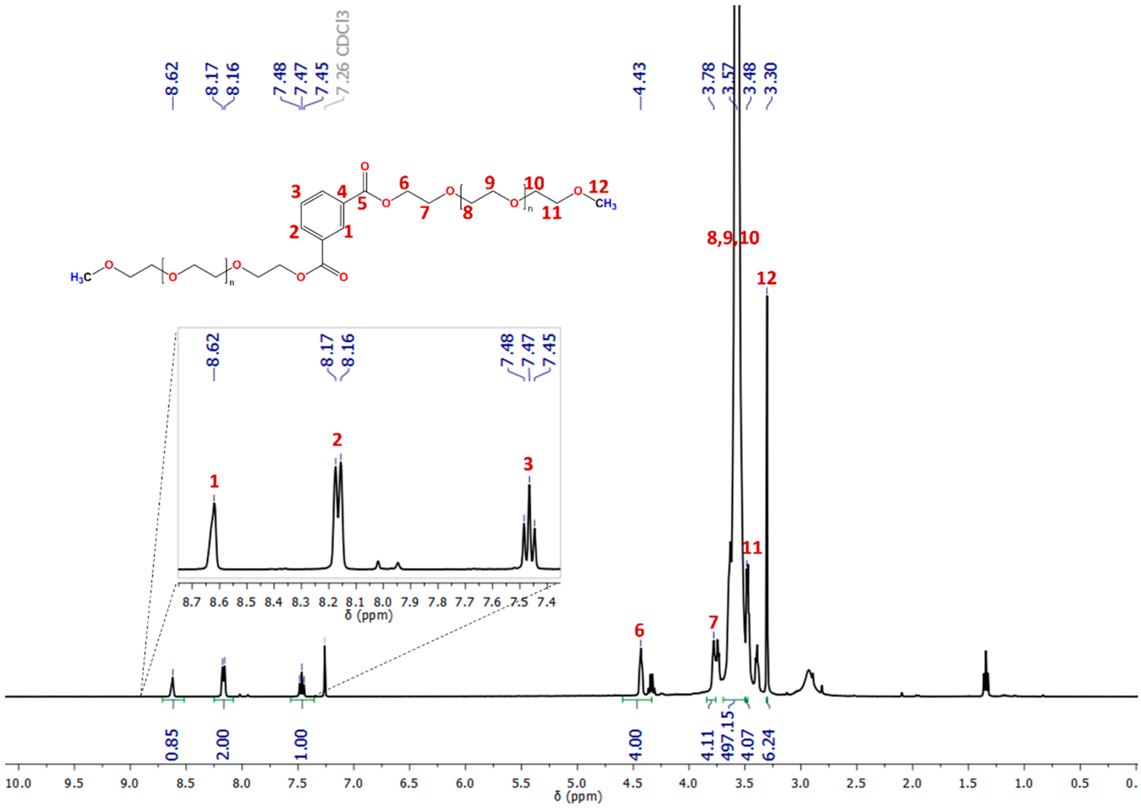

8.62
8.17
8.16
7.48
7.47
7.45
7.26 CDCl3
4.43
3.78
3.57
3.48
3.30
8,9,10
12
11
7
6
0.85
2.00
1.00
4.00
4.11
497.15
4.07
6.24
δ (ppm)

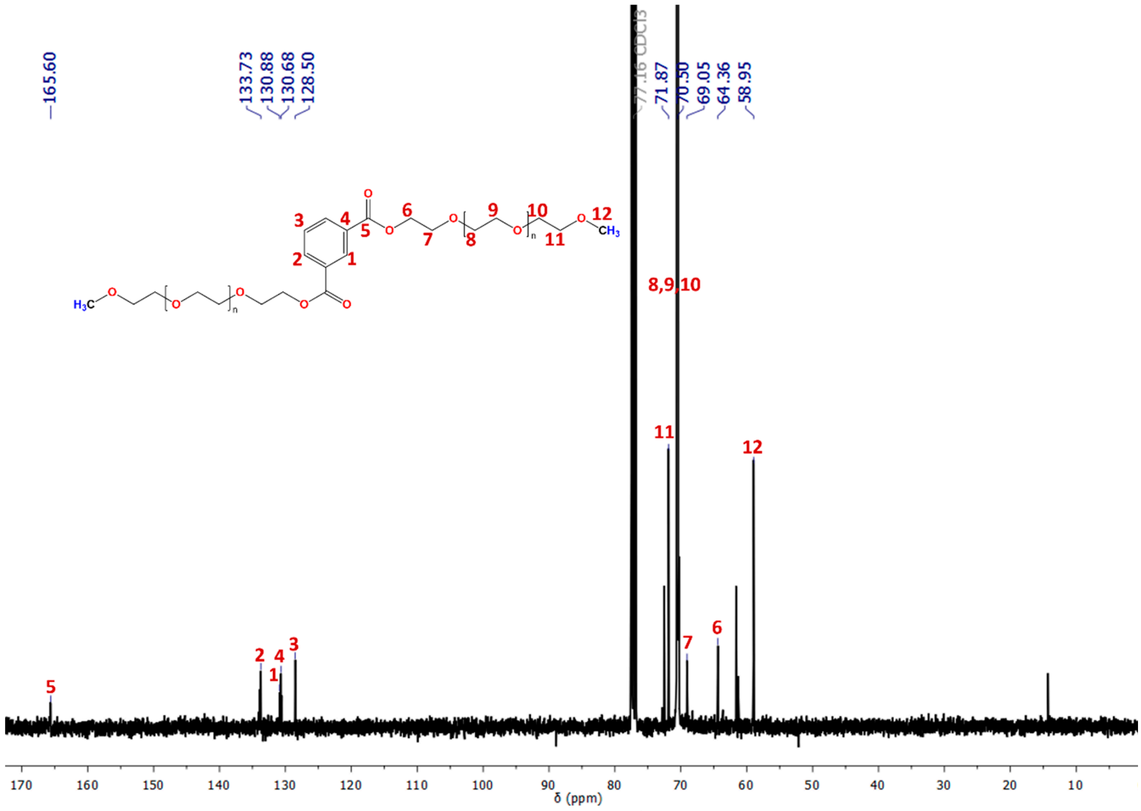

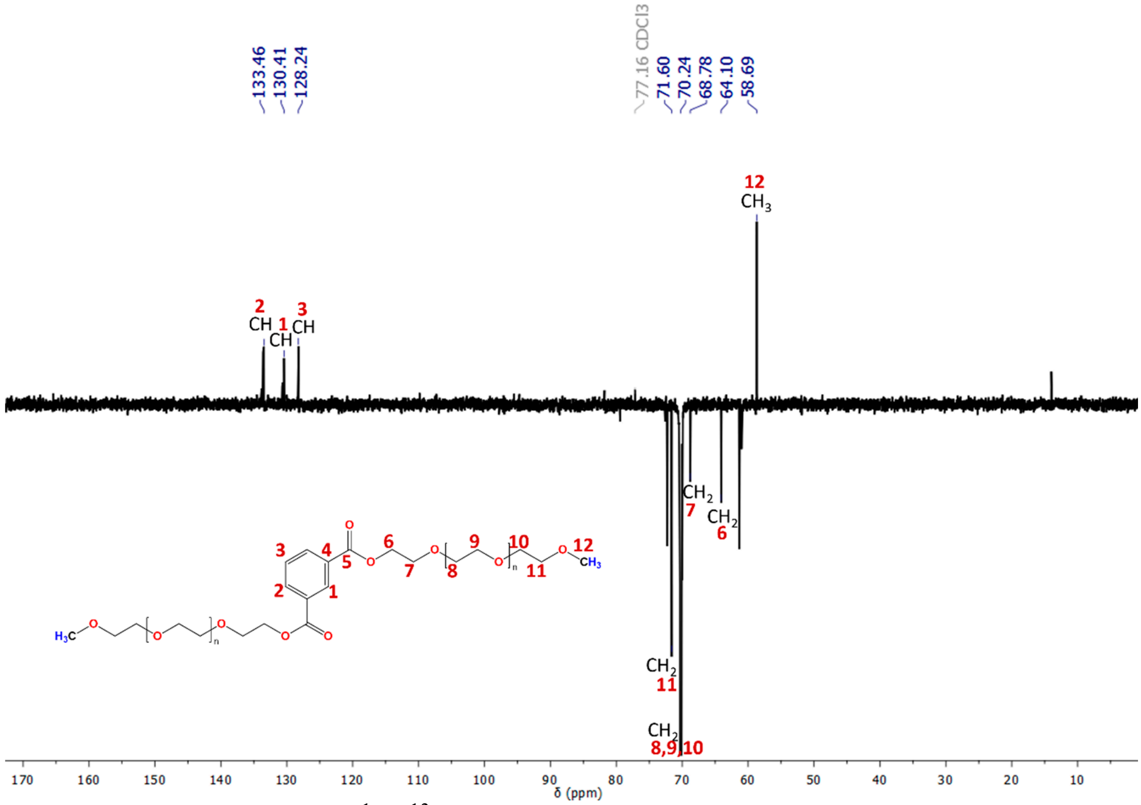

Figure S10. Infrared and ^{1}H, ^{13}C and DEPT 135° NMR spectra of IPC-MPEG2000.

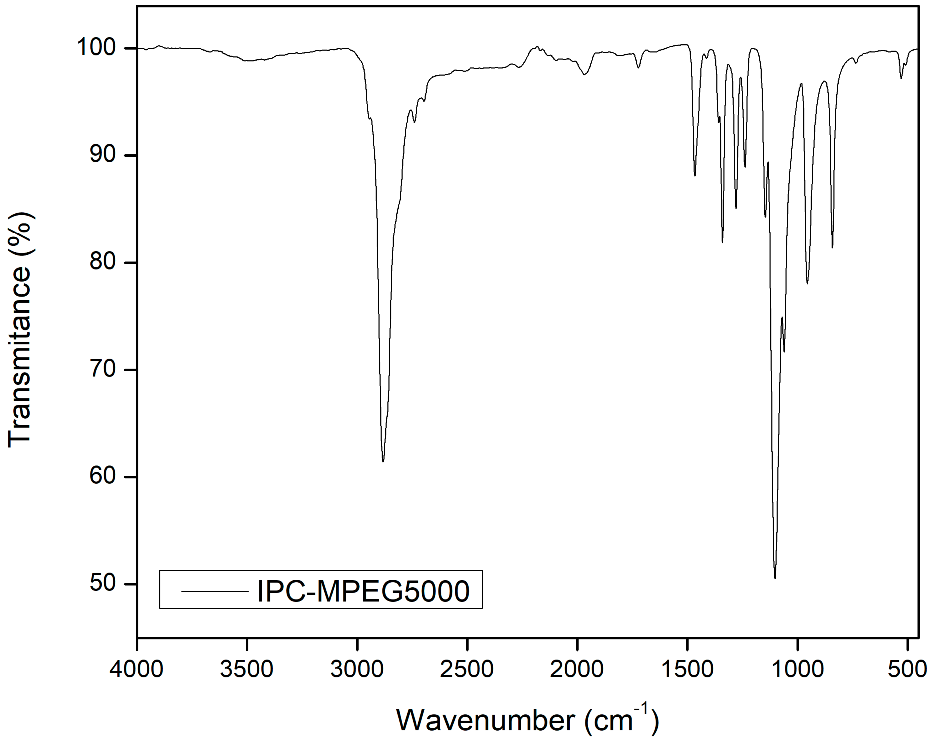

Transmitance (%)
Wavenumber (cm-1)
IPC-MPEG5000

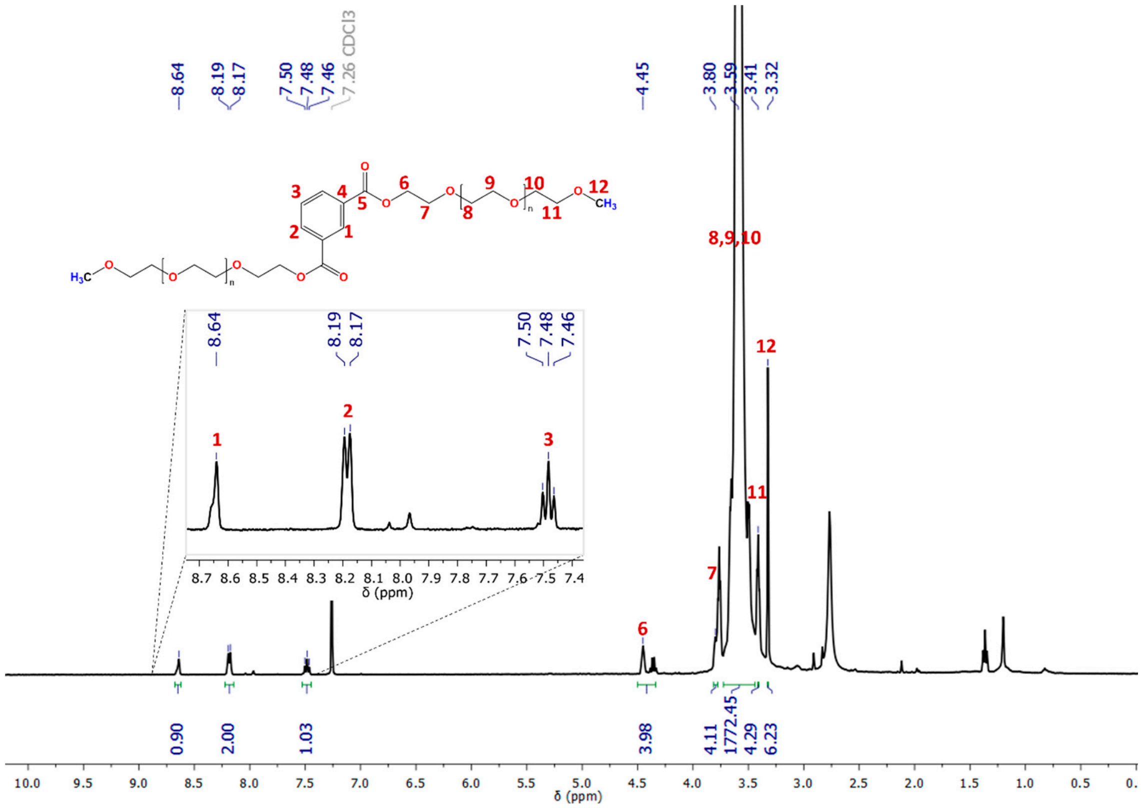

8.64
8.19
8.17
7.50
7.48
7.46
7.26 CDCl3
4.45
3.80
3.59
3.41
3.32
8,9,10
12
11
7
6
0.90
2.00
1.03
3.98
4.11
1772.45
4.29
6.23
δ (ppm)

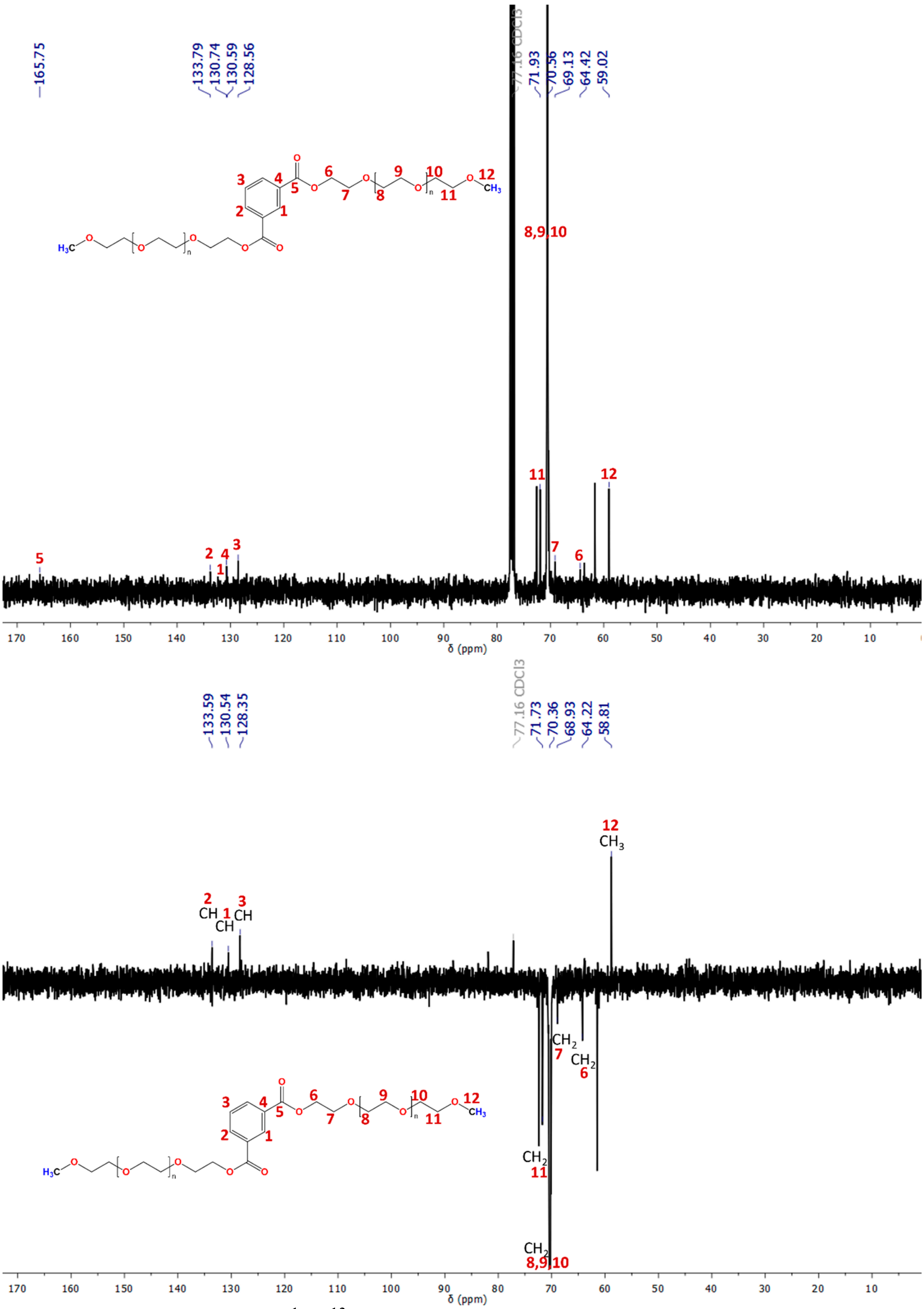

Figure S11. Infrared and ^{1}H, ^{13}C and DEPT 135° NMR spectra of IPC-MPEG5000.

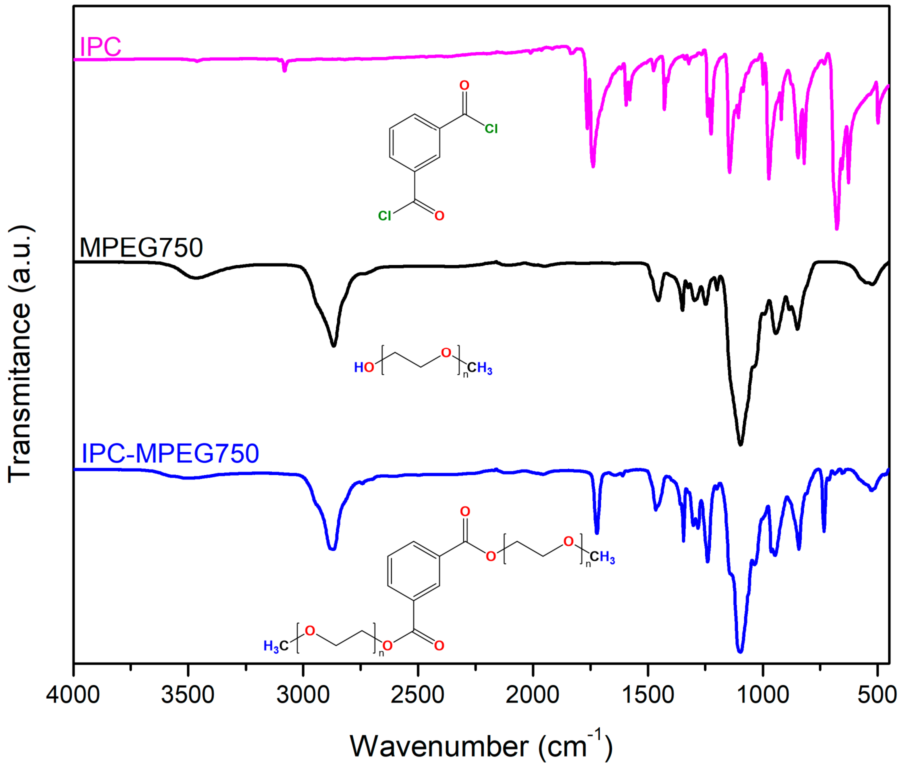

Figure S12. Comparison of IPC, MPEG 750 and IPC-MPEG750 infrared spectra.

Table S2. Theoretical hydrogen counts (I_t) calculated in the repeating unit, and hydrogen counts obtained by ^{1}H NMR in the IPC-MPEG series.

Signal number	δ (ppm) and integral (*I*)	PCMs				
		IPC-MPEG350	IPC-MPEG550	IPC-MPEG750	IPC-MPEG2000	IPC-MPEG5000
1	δ	8.61	8.65	8.64	8.62	8.64
	I	1	1	1	1	1
2	δ	8.15	8.19	8.18	8.17	8.18
	I	2	2	2	2	2
3	δ	7.44	7.78	7.48	7.47	7.48
	I	1	1	1	1	1
6	δ	4.41	4.45	4.45	4.43	4.45
	I	4	4	4	4	4
7	δ	3.76	3.80	3.79	3.78	3.80
	I	4	4	4	4	4
8,9,10	δ	3.55	3.59	3.58	3.57	3.59
	I	50	89	125	497	1773
11	δ	3.45	3.49	3.48	3.48	3.41
	I	4	4	4	4	4
12	δ	3.28	3.32	3.32	3.30	3.32
	I	6	6	6	6	6
Integral	$\sum_6^{11} I_n$	62	101	137	509	1785
	I_t	58	94	130	357	902

δ: Chemical shift in ^{1}H NMR spectrum, *I*: Integral obtained by ^{1}H NMR, I_t: Integral obtained by Eq. (1) and $\sum_6^{11} I_n$: Summation of the integrals of signals 6 to 11 obtained by ^{1}H NMR.
